# Argonaute 2 and nasopharyngeal carcinoma: a genetic association study and functional analysis

**DOI:** 10.1186/s12885-015-1895-4

**Published:** 2015-11-06

**Authors:** Peiyao Li, Jinfeng Meng, Yun Zhai, Hongxing Zhang, Lixia Yu, Zhifu Wang, Xiaoai Zhang, Pengbo Cao, Xi Chen, Yuqing Han, Yang Zhang, Huipeng Chen, Yan Ling, Yuxia Li, Ying Cui, Jin-Xin Bei, Yi-Xin Zeng, Fuchu He, Gangqiao Zhou

**Affiliations:** 1 State Key Laboratory of Proteomics, Beijing Proteome Research Center, Beijing Institute of Radiation Medicine, No. 27, Taiping Road, Haidian District, Beijing, 100850 P.R. China; 2 Chinese Academy of Medical Sciences & Peking Union Medical College, Institute of Basic Medical Sciences, Beijing, P.R. China; 3 Laboratory of Microbial Genomics, Beijing Institute of Biotechnology, Beijing, P.R. China; 4 Affiliated Cancer Hospital of Guangxi Medical University, Nanning, P.R. China; 5 State Key Laboratory of Oncology in Southern China, Department of Experimental Research, Sun Yat-sen University Cancer Center, Guangzhou, P.R. China

**Keywords:** *AGO2*, Polymorphism, Nasopharyngeal carcinoma, Lymph node metastasis

## Abstract

**Background:**

Argonaute 2 (AGO2), a central component of RNA-induced silencing complex, plays critical roles in cancer. We examined whether the single nucleotide polymorphisms (SNPs) of *AGO2* were related to the risk of nasopharyngeal carcinoma (NPC).

**Methods:**

Twenty-five tag SNPs within *AGO2* were genotyped in Guangxi population consisting of 855 NPC patients and 1036 controls. The SNPs significantly associated with NPC were further replicated in Guangdong population consisting of 996 NPC patients and 972 controls. Functional experiments were conducted to examine the biologic roles of *AGO2* in NPC.

**Results:**

A significantly increased risk of advanced lymph node metastasis of NPC was identified for the *AGO2* rs3928672 GA + AA genotype compared with GG genotype in both the Guangxi and Guangdong populations (combined odd ratio = 2.08, 95 % confidence interval = 1.44-3.01, *P* = 8.60 × 10^−5^). Moreover, the AGO2 protein expression levels of rs3928672 GA + AA genotype carriers were higher than the GG genotype carriers in the NPC tissues (*P* = 0.041), and AGO2 was significantly over-expressed in NPC tissues compared with non-cancerous nasopharyngeal tissues (*P* = 0.011). In addition, *AGO2* knockdown reduced cell proliferation, induced apoptosis, and inhibited migration of NPC cells. Furthermore, gene expression microarray showed that genes altered following *AGO2* knockdown were clustered in tumorigenesis and metastasis relevant pathways.

**Conclusions:**

Our findings suggest that the genetic polymorphism in *AGO2* may be a risk factor for the advanced lymph node metastasis of NPC in Chinese populations, and *AGO2* acts as an oncogene in the development of NPC.

**Electronic supplementary material:**

The online version of this article (doi:10.1186/s12885-015-1895-4) contains supplementary material, which is available to authorized users.

## Background

Nasopharyngeal carcinoma (NPC) is an epithelial malignancy with striking racial and geographic distribution differences. It is particularly prevalent among populations from southern China, Southeast Asia, northern Africa and Alaska. These incidence rates are approximately 100-fold higher than in the Caucasian populations [1]. Several environmental factors, including infection with the Epstein-Barr virus (EBV), long-term cigarette smoking, occupational exposure to formaldehyde, and various dietary factors, have been reported to confer the risk of developing NPC [2]. Furthermore, numerous genetic linkage and association studies have reported a few genes contributing to the risk of this malignancy [3, 4]. The identification of susceptibility genes contributing to NPC would assist in predicting individual and population risk of NPC development and would help to clarify the pathogenesis relevant to this disorder.

Argonaute 2 (AGO2), a member of the Argonaute protein family, can bind microRNAs (miRNAs) or short interfering RNAs (siRNAs) and mediate the repression of specific target RNAs either by degrading RNA or inhibiting translation [5]. Of the four human AGO proteins, AGO2 is the only member with intrinsic endoribonuclease activity and essential non-redundant slicer-independent function within the mammalian miRNA pathway [6]. Recently, alterations (genomic amplifications and/or over-expression) of the *AGO2* gene have been extensively described in a variety of cancers [7–11], and these alterations have been shown to be linked with an increased metastasis [11–13] and poorer prognosis [12]. Further studies indicated that the AGO2 is involved in several steps of cancer development, including cell proliferation, differentiation, apoptosis, migration and invasion [8–10, 14, 15]. With regard to NPC, the elevated mRNA expressions of *AGO2* were observed in tumor tissues and latent membrane protein 1 (LMP1)-positive tumors compared with normal adjacent nasopharyngeal epithelium tissues and LMP1-negative tumors, respectively [16]. Taken together, these studies suggest that the AGO2 may play crucial roles in the cancer development and progression.

Theoretically, the genetic variants such as single nucleotide polymorphisms (SNPs) within the *AGO2* gene, which may alter the expression of *AGO2* and then influence the miRNAs processing and function, could result in genotype-dependent differences in risk of cancers. Indeed, there is increasing evidence that the genetic variants in the *AGO2* gene are associated with the risk or development of several cancers, including breast cancer [17, 18] and malignant peripheral nerve sheath tumor [19]. The role of genetic variants within *AGO2* in NPC, however, has never been specifically investigated. In the present study, we examined whether the genetic variation in the *AGO2* gene affect the risk or severity of NPC in the Chinese populations. We also evaluated the biologic roles of *AGO2* in the development of NPC by functional assays.

## Methods

### Study population

This study consisted of two populations of patients with NPC and control subjects resided in Guangxi and Guangdong province, respectively, both of which were well-known high-risk regions for NPC located in southern China (Additional file 1: Table S1). The Guangxi population, which contained 855 incident patients with NPC and 1036 controls, has been described in detail previously [20]. Briefly, all subjects were unrelated ethnic adult Chinese and residents in Nanning city (Nanning, China) and the surrounding regions. All patients were newly diagnosed and pathologically confirmed, and were consecutively recruited between September 2003 and January 2008 at the Guangxi Cancer Hospital (Nanning, China). Patients that received chemotherapy or radiotherapy prior to surgery or had other type of cancer were excluded from this study. Tumor staging was performed according to the tumor-node-metastasis (TNM) classification by the 1997 American Joint Committee on Cancer (AJCC) system. All TNM classifications were determined by senior pathologists of the hospital on the basis of the postoperative histopathologic examination. All the controls were recruited in the same regions during the same time that the NPC cases were collected. The selection criteria for the controls included no individual history of cancer and frequency matching to the cases on sex and age (± 5 years). The Guangdong population, which contained 997 NPC patients and 972 controls, has been described in detail previously [3]. Briefly, all subjects were unrelated ethnic adult Chinese and residents in Guangdong province. All patients were consecutively recruited between October 2005 and October 2007 from Sun Yat-sen University Cancer Center (SYSUCC) in Guangzhou city (Guangzhou, China). All patients were histopathologically diagnosed by at least two pathologists according to the World Health Organization (WHO) classification. During the same period, control subjects were recruited from physical examination centers of several large comprehensive hospitals in local communities in Guangdong and were frequency matched to the cases by age (± 5 years), sex, geographic region and ethnicity. All the subjects were interviewed for collection of personal information on demographic factors, medical history, cigarette smoking and alcohol use via structured questionnaire. This study was approved by the Ethics Committee of Beijing Institute of Radiation Medicine (Beijing, China). At recruitment, the written informed consent was obtained from all the participants involved in this study.

From the Guangxi population of 855 patients with incident NPC, 37 patients who had undergone resection before receiving any further treatment at Guangxi Cancer Hospital were selected, and primary NPC biopsies were collected from them (Additional file 1: Table S2). The histological type of all tumor tissues was poorly differentiated squamous cell carcinoma (SCC). Histological non-cancerous nasopharyngeal epithelium tissues were collected from 18 of the 1036 control subjects (Additional file 1: Table S2). All the tissues were fixed in paraformaldehyde, embedded in paraffin wax, and prepared for subsequent immunohistochemical staining.

### SNP selection and genotyping

Twenty-five SNPs in the *AGO2* gene were selected for genotyping in our study (Table 1). These SNPs were chosen on the basis of previous reports of their association with the risk for cancer, or with potential function, and a comprehensive tag SNP approach. Two SNPs (rs2292779 and rs7005286) significantly associated with cancer in previous studies [18, 19] were directly selected in our study. Two SNPs (rs4961280 and rs11996715) located in the promoter region (−1809A/C and −1686 A/C) of *AGO2*, were selected as they could alter the expression of *AGO2*. Tag SNPs of the *AGO2* gene were selected from the Han Chinese in Beijing, China (CHB) HapMap database (HapMap release 27, February 2009) using HaploView (pairwise *r*^2^ > 0.8) [21]. To ensure enough statistical power, a value of 0.10 of minor allele frequency (MAF) was set as the threshold value of inclusion in this study. Finally, 25 tag SNPs were selected to capture the *AGO2* gene.

**Table 1:** Positions and frequencies of SNPs within *AGO2* gene

No.	SNPs^a^	Alleles (Minor/Major)	Frequencies^b^	Positions^c^	Regions
1	rs4961280	C/A	0.111	−1809	Promoter
2	rs11996715	C/A	0.430	−1686	Promoter
3	rs4961226	G/T	0.400	3050	Intron 1
4	rs10088596	C/T	0.453	5311	Intron 1
5	**rs883596**	G/C	0.105	9957	Intron 1
6	rs13261055	A/G	0.386	16208	Intron 1
7	rs7001653	G/A	0.444	24567	Intron 1
8	rs7819727	A/G	0.452	48394	Intron 1
9	rs7009635	T/C	0.367	50724	Intron 2
10	rs7005286	C/T	0.337	51145	Intron 2
11	rs11776034	C/G	0.211	53712	Intron 2
12	rs3735805	C/T	0.356	68083	Intron 3
13	**rs2977490**	A/G	0.453	72247	Intron 3
14	rs2292773	C/T	0.278	73107	Intron 4
15	rs3928672	G/A	0.222	73438	Intron 4
16	rs2271735	G/T	0.178	76295	Intron 6
17	**rs2977481**	G/C	0.183	83748	Intron 10
18	rs2292779	G/C	0.244	84394	Intron 11
19	rs2944764	C/T	0.239	84576	Intron 11
20	rs11166983	G/A	0.278	87519	Intron 11
21	rs12542354	C/T	0.239	89462	Intron 13
22	rs13252337	A/G	0.186	96222	Intron 16
23	rs2977469	G/A	0.465	96314	Intron 16
24	rs2977464	C/T	0.333	100412	Intron 16
25	rs2977462	T/C	0.467	102467	Intron 16

SNPs in bold were not in Hardy-Weinberg equilibrium (HWE) (rs883596 and rs2977490, both *P* < 0.05) or failed genotyping (rs2977481) in the Guangxi population

^a^dbSNP ID number

^b^Minor allele frequencies in HapMap-HCB (HCB, unrelated Han Chinese in Beijing, China)

^c^The position of the SNPs is relative to the first nucleotide of the open reading frame of the *AGO2* gene, referred to as accession number NT_008046.15

All SNPs were genotyped using GenomeLab SNPstream Genotyping System according to the manufacturer’s instructions (Beckman Coulter, USA) as previously described [22]. This platform uses a single base pair extension reaction to incorporate two-color fluorescence terminal nucleotides which are detected by a specialized imager. Details of the primer sequences are listed in Additional file 1: Table S3. The genotype data were analyzed by GenomeLab SNPstream version 2.2 software (Beckman Coulter, USA). To ensure genotyping quality, we genotyped 20 randomly selected duplicate samples and 4 blanks in each 384-well plate and obtained a concordance rate of >99 %.

### Immunohistochemistry in NPC tissues and non-cancerous nasopharyngeal tissues

The paraformaldehyde-fixed and paraffin-embedded NPC tissues (n = 37) and non-cancerous nasopharyngeal tissues (n = 18) (Additional file 1: Table S2) were analyzed for protein expression of AGO2. Two slides from each biopsy were stained with hematoxylin and eosin for routine histological evaluation. Histologic slides with tissue sections were subjected to immunohistochemistry (IHC) as previously described [20, 22], using the primary antibody raised against AGO2 (diluted 1:200; ab57113, Abcam, UK). Negative controls and positive controls were performed at the same time. Photographs were taken (BX51 microscopic/Digital Camera System; Olympus) for study comparison. The IHC signals were scored as previously described [20, 22] by two pathologists (Wu J and Huang W) who did not have knowledge of ligand-binding assay results or patient outcome. Briefly, a proportion score was assigned representing the estimated proportion of positive staining tumor cells (0, none; 1, < 1/100; 2, 1/100 to < 1/10; 3, 1/10 to < 1/3; 4, 1/3 - 2/3; 5, > 2/3). Average estimated intensity of staining in positive cells was assigned an intensity score (0, none; 1, weak; 2, intermediate; 3, strong). The two parameters were combined and resulting in an overall score (0 or 2–8). The scores were classified into three groups: group 1 (score 0, negative expression), group 2 (score 2–4, low expression) and group 3 (score 5–8, high expression). A total of five fields per slide were selected, counted, and averaged.

### *AGO2* knockdown in NPC cell line

The human nasopharyngeal carcinoma cell line CNE2Z was obtained from the Peking Union Medical College (Chinese Academy of Medical Science, China) and maintained in RPMI 1640 (Gibco-BRL, USA) supplemented with 10 % fetal bovine serum (HyClone, USA) and 1 % penicillin/streptomycin (Gibco-BRL, USA) at 37 °C under a 5 % CO_2_ atmosphere. The *AGO2* specific shRNA expression vectors and the scrambled ineffective shRNA cassette (as the negative control) in the pGPU6/GFP/Neo plasmid were purchased from GenePharma (China). The sequences of the three shRNAs directed against *AGO2* are as follows: sh1: 5’-GCAAGGATCGCATCTTCAAGG-3’; sh2: 5’-GGTCTAAAGGTGGAGATAAGG-3’; and sh3: 5’-GCCTGAAGATCAACGTCAAGC-3’. The sequence of control shRNA is 5’-GTTCTCCGAACGTGTCACGT-3’. A mixture of constructs including three *AGO2* shRNAs or the control shRNA construct was transfected into CNE2Z cells using Lipofectamine 2000 (Invitrogen, USA). The efficiency of RNA interfering was assessed by western blot analysis.

### Western blot

Cells were lysed in Laemmli sample buffer (Bio-Rad, USA) with an EDTA-free, protease inhibitor cocktail (Roche, USA). Proteins at the same amount were separated by SDS-PAGE and transferred onto polyvinylidene difluoride (PVDF) membranes (Millipore, USA). After probing with primary and secondary antibody, antigen-antibody complex was visualized by enhanced chemiluminescence-plus reagent (Pierce Biotechnologies, USA). For AGO2, mouse anti-AGO2 monoclonal antibody (diluted 1:1000; ab57113, Abcam, UK) was used. As an internal control, mouse anti-β-actin monoclonal antibody (diluted 1:1000; sc-130301, Snata cruz, USA) was used.

### Cell proliferation, apoptosis and migration assays

Cell proliferation was evaluated by measuring cell viability with Cell Counting Kit-8 (CCK-8) assay (Beyotime Inst Biotech, China) according to the manufacturer’s instructions. Cells (2 × 10^3^ cells per well) were plated in 96-well plates in triplicate. CCK-8 was added to each well at a final concentration of 10 % at different time points (24, 48, 72 and 96 h) and incubation continued at 37 °C for 50 min. Subsequently, the absorbance of the samples was measured at 450 nm using a Multiskan MK3 microplate reader (Thermo Labsystems, USA) to calculate the numbers of viable cells in each well.

Apoptosis was detected by flow cytometry using the Annexin V-APC/7-AAD Apoptosis Detection Kit (KeyGEN, China). Briefly, cells were harvested, washed, resuspended in the staining buffer, and doubly stained with annexin-V and 7-amino-actinomycin D (7-AAD). For each experiment, 5 × 10^4^ cells were analyzed using FACSCalibur and CellQuest software (BD Biosciences, USA). The Annexin V-positive cells were regarded as apoptotic cells.

Ability of cell migration was evaluated by Transwell assay (Corning Inc, USA) according to the manufacturer’s instructions. Cells (1.5 × 10^5^ cells per well) in 300 μl serum-free medium were placed in the upper chamber of the transwell, whereas the lower chamber was loaded with 500 μl medium containing 20 % fetal bovine serum. After 16 h of incubation, cells that migrated to the lower chamber were fixed and stained with crystal violet. The number of cells was counted in five random microscopic fields (magnification 400 ×).

### Genome-wide expression microarray and pathway analysis

Total RNA was isolated for microarray analysis from three biological replicates of cells transfected with *AGO2* shRNAs or control shRNA using TRIzol reagent (Invitrogen, USA) according to the manufacturer’s instructions. All samples with an RNA integrity number (RIN) ≥ 8.0 and A260/A280 ratio 1.8-2.1 were considered suitable for microarray analysis. The Affymetrix GeneChip® Human Gene U133 Plus 2.0 Arrays were used for genome-wide expression profiling assay, which was performed by CapitalBio Corporation (Beijing, China) according to the manufacturer’s instructions (Affymetrix, USA). Raw feature data from microarrays were subsequently corrected for background and normalized, and the log_2_ intensity expression summary values for each probe set were calculated using Robust Multiplechip Average (RMA). Significantly altered genes following *AGO2* knockdown were analyzed by significance analysis of microarray (SAM, version 3.01), and the *P* values of the *t* test were calculated for each gene. Multiple hypothesis testing was performed to calculate the false discovery rates (FDRs) through the Bioconductor package Qvalue (http://www.bioconductor.org). Significantly altered genes following *AGO2* knockdown were investigated for biological processes and signaling pathways using the cytoscape plug-in of Reactome FI [23].

### Statistical analysis

Genotype and allele frequencies of *AGO2* polymorphisms were determined by direct counting, and departures from Hardy-Weinberg equilibrium (HWE) were tested using the random-permutation procedure implemented in the Arlequin package (http://cmpg.unibe.ch/software/arlequin3/). Multivariate logistic regression analysis was done to evaluate whether there were genetic associations between the *AGO2* polymorphisms and risk and severity of NPC. The *P* values, odds ratios (ORs), and 95 % confidence intervals (CIs) were calculated and adjusted for sex, age, status of smoking and drinking, smoking level, family history and nationality where it was appropriate. To assess the probability of a spurious association due to multiple comparisons, the online software SNPSpD (http://gump.qimr.edu.au/general/daleN/SNPSpD) was used to calculate the correction factor for multiple testing in gene unit [24]. A *P* value of 0.0036 (0.05/14.01; correction factor = 14.01) was used as the criterion of statistical significance in Guangxi population. Association analysis was performed by SNPStats (http://bioinfo.iconcologia.net/snpstats/start.htm). The power for our genetic association study was calculated using the Power for Genetic Association Analyses (PGA) [25]. Differences of the protein expression levels detected by IHC between the NPC tissues and non-cancerous nasopharyngeal tissues, and between the rs3928672 GG genotype and A allele (GA + AA genotype) carriers were assessed by a Wilcoxon signed-ranks test and logistic regression analysis, respectively. Unpaired *t* test was used to analyze the results of cell proliferation, apoptosis and migration assays. A *P* value of 0.05 was used as the criterion of statistical significance, and all statistical tests were two sided. These analyses were performed using SPSS software (Version 17.0; SPSS Inc., USA).

## Results

### Characteristics of the study population

For the Guangxi population, the selected demographic and clinical characteristics were described in detail previously (Additional file 1: Table S1) [20]. In the Guangdong population, there was no significant difference between the patients and control subjects in terms of mean age, sex, status of smoking and drinking; while more heavier smokers (≥ 24 pack-years) were present in the cases compared with controls (*χ*^2^ = 13.42, *P* = 2.48 × 10^−4^). According to the TNM systems, 3.6 %, 14.9 %, 50.5 % and 25.4 % of patients had stage I, II, III, and IV disease, respectively (Additional file 1: Table S1).

### Genetic association between SNPs of the *AGO2* gene and NPC

The 25 SNPs were initially genotyped in the Guangxi population. We succeeded in genotyping 24 (with the exception of rs2977481) out of 25 SNPs using the SNPstream assay. Of these 24 SNPs, 2 (rs883596 and rs2977490) were not in HWE (both *P* < 0.05). We therefore selected the remaining 22 SNPs for the subsequent analyses.

The genotyping results for the 22 SNPs are shown in Additional file 1: Table S4. On the basis of logistic regression analysis with adjustment for sex, age, status of smoking and drinking, smoking level, family history and nationality, only one SNP, i.e. rs12542354, was significantly associated with the susceptibility to NPC in the Guangxi population after correction for multiple comparisons (OR = 1.32, 95 % CI = 1.13-1.54, *P* = 0.00040; Additional file 1: Table S4). Then, the rs12542354 was further genotyped in the Guangdong population. However, the initial association between the rs12542354 and risk of NPC in the Guangxi population was not validated in the Guangdong population (Additional file 1: Table S4).

Then, we assessed the effects of the *AGO2* polymorphisms on the severity of NPC (as measured by TNM staging system) in the Guangxi population. After adjustment for sex, age, status of smoking and drinking, smoking level, family history and nationality, logistic regression analysis revealed that significant association with the advanced local tumor invasion of NPC was observed with the rs2271735 (Additional file 1: Table S5). In the meantime, the rs2977469 and rs3928672 were significantly associated with the advanced lymph node involvement of NPC (Additional file 1: Table S6). The associations remained significant even after correction for multiple comparisons. To confirm the initial associations of these three SNPs (rs2271735, rs2779249 and rs3928672) with the NPC severity, the Guangdong population, composed of 997 patients with NPC, were also genotyped (Additional file 1: Table S5 and Additional file 1: Table S6). Only rs3928672 was validated in the Guangdong population. In the Guangxi population, the patients bearing rs3928672 A allele (GA + AA genotype) had a significantly increased frequent of involvement of lymph node compared with ones bearing GG genotype (N3 vs. N0 + N1 + N2; OR = 2.47, 95 % CI = 1.47-4.13, *P* = 0.00030; Table 2). Consistently, patients with the rs3928672 A allele (GA + AA genotype) had an advanced lymph node metastasis (N3 vs. N0 + N1 + N2; OR = 1.75, 95 % CI = 1.03-2.98, *P* = 0.034; Table 2) compared to ones with the GG genotype in the Guangdong population. The distribution of rs3928672 genotype frequencies is not different between the four N classification sub-populations. Therefore, we pooled the two case-control studies from the Guangxi and Guangdong populations, and found that the association of rs3928672 with the susceptibility to advanced lymph node metastasis was stronger (N3 vs. N0 + N1 + N2; OR = 2.08, 95 % CI = 1.44-3.01, *P* = 8.60 × 10^−5^; Table 2). A meta-analysis also confirmed the strong evidence of association of rs3928672 with the advanced lymph node metastasis (N3 vs. N0 + N1 + N2; OR = 2.09, 95 % CI = 1.44-3.03, *P* = 9.64 × 10^−5^; Table 2). There was little evidence of heterogeneity among sample sets (*P*_heterogeneity_ = 0.36).

**Table 2:** Genetic associations of rs3928672 with the lymph node metastasis of NPC

Populations	Genotypes	N classification, *n* (%)				OR (95 % CI)^a^	*P* value^a^
		N0	N1	N2	N3		
Guangxi		(*n* = 177)	(*n* = 406)	(*n* = 180)	(*n* = 78)		
	GG	91 (51.4)	187 (46.1)	95 (52.8)	22 (28.2)	1.00 (reference)	
	GA	72 (40.7)	177 (43.6)	69 (38.3)	47 (60.3)	2.50 (1.47-4.25)	0.0010
	AA	14 (7.9)	42 (10.3)	16 (8.9)	9 (11.5)	2.31 (1.01-5.27)	0.046
	GA + AA	86 (48.6)	219 (53.9)	85 (47.2)	56 (71.8)	2.47 (1.47-4.13)	0.00030
Guangdong		(*n* = 226)	(*n* = 372)	(*n* = 260)	(*n* = 65)		
	GG	123 (54.4)	184 (49.5)	134 (51.5)	25 (38.5)	1.00 (reference)	
	GA	87 (38.5)	152 (40.8)	105 (40.4)	37 (56.9)	1.95 (1.14-3.34)	0.015
	AA	16 (7.1)	36 (9.7)	21 (8.1)	3 (4.6)	0.79 (0.23-2.72)	0.71
	GA + AA	103 (45.6)	188 (50.5)	126 (48.5)	40 (61.5)	1.75 (1.03-2.98)	0.034
Pooled		(*n* = 403)	(*n* = 778)	(*n* = 440)	(*n* = 143)		
	GG	214 (53.1)	371 (47.7)	229 (52.0)	47 (32.9)	1.00 (reference)	
	GA	159 (39.5)	329 (42.3)	174 (39.5)	84 (58.7)	2.22 (1.51-3.20)	4.07 × 10^−5^
	AA	30 (7.4)	78 (10.0)	37 (8.5)	12 (8.4)	1.54 (0.79-2.99)	0.21
	GA + AA	189 (46.9)	407 (52.3)	211 (48.0)	96 (67.1)	2.08 (1.44-3.01)	8.60 × 10^−5^
Overall^b^		(*n* = 403)	(*n* = 778)	(*n* = 440)	(*n* = 143)		
	GG	214 (53.1)	371 (47.7)	229 (52.0)	47 (32.9)	1.00 (reference)	
	GA	159 (39.5)	329 (42.3)	174 (39.5)	84 (58.7)	2.21 (1.52-3.23)	3.82 × 10^−5^
	AA	30 (7.4)	78 (10.0)	37 (8.5)	12 (8.4)	1.66 (0.83-3.29)	0.15
	GA + AA	189 (46.9)	407 (52.3)	211 (48.0)	96 (67.1)	2.09 (1.44-3.03)	9.64 × 10^−5^

Note: Guangxi and Guangdong populations consist of 855 and 962 patients with known lymph node involvement (N classification) stage, respectively. Due to DNA quality and/or quantity, the actual sample sizes were 841 and 923 patients in the Guangxi and Guangdong population, respectively

*Abbreviations: N* lymph node involvement, *OR* odds ratio, *CI* confidence interval

^a^In the Guangxi population, the ORs, 95 % CIs and *P* values were calculated for N3 vs. N0 + N1 + N2 and adjusted for sex, age, status of smoking and drinking, smoking level, family history and nationality. In the Guangdong population, the ORs, 95 % CIs and *P* values were calculated for N3 vs. N0 + N1 + N2 and adjusted for sex, age, status of smoking and drinking, and smoking level. In the pooled population, the ORs, 95 % CIs and *P* values were calculated for N3 vs. N0 + N1 + N2 and adjusted for sex, age, status of smoking and drinking, and smoking level. Another confounding factor, native place, was also adjusted in the pooled population

^b^A meta-analysis combining two independent case–control studies. The *P* values for heterogeneity among sample sets are 0.52 for GA vs. GG group, 0.18 for AA vs. GG and 0.36 for GA + AA vs. GG group, respectively

The association between the rs3928672 A allele (GA + AA genotype) and advanced lymph node metastasis of NPC were further examined with stratification by age, sex, status of smoking and drinking, smoking level, family history and nationality (Additional file 1: Table S7). A significantly increased risk of lymph node metastasis of NPC was found in the heavier smokers (≥ 24 pack-years) compared with the lighter smokers (*P* = 0.022, test for homogeneity). However, the smoking level had no modification effect on the severity of NPC related to the rs3928672 in the Guangdong population.

The power for this association study was calculated using the PGA program [25], based on our sample sizes in the Guangxi population (78 patients with N3 stage vs. 763 patients with N0 + N1 + N2 stage), the minor A allele frequency of 31.3 %, and an OR of 2.47. By this calculation, the power of our initial association study in the Guangxi population to detect rs3928672 was estimated to be 44.0 %.

### Effect of the rs3928672 on AGO2 protein expression

**Fig. 1:**
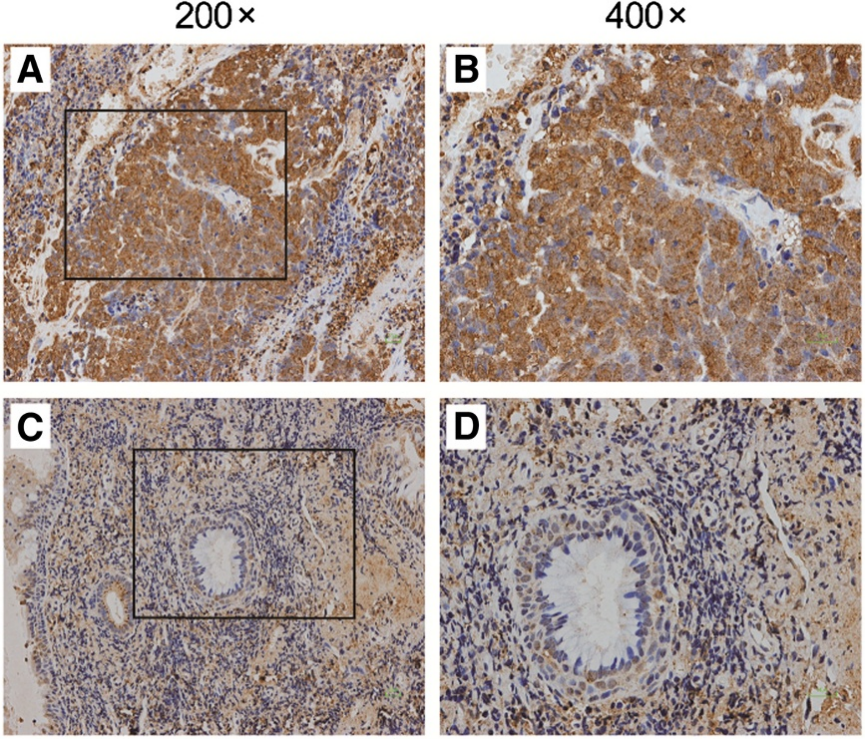
Protein expression level of AGO2 by immunohistochemical staining in representative NPC tissues and non-cancerous nasopharyngeal tissues. Panels **a** and **b** NPC tissues; Panels **c** and **d** non-cancerous nasopharyngeal tissues. Images in the box (left, magnification 200 ×) were enlarged and shown in the right (magnification 400 ×). The AGO2 protein was found to locate in the cytoplasm of malignant cells

We further investigated the protein expression level of AGO2 in NPC tissues and non-cancerous nasopharyngeal tissues by IHC assay. Consistent with a previous report [12], the AGO2 protein was mainly accumulated in the cytoplasm of malignant cells. We detected high expression of AGO2 in the 32 of 37 NPC tissues (86.5 %), but in the 10 of 18 non-cancerous nasopharyngeal tissues (55.5 %) (*χ*^2^ = 6.42, *P* = 0.011; Fig. 1 and Additional file 1: Table S8). Furthermore, there was significant association between the rs3928672 genotypes and expression level of AGO2 protein in the NPC tissues, with the A allele (GA + AA genotype) carriers having higher AGO2 expression than the GG genotype carriers (*P* = 0.041; Additional file 1: Table S8).

### Effect of *AGO2* knockdown on cell proliferation, apoptosis and migration in vitro

**Fig. 2:**
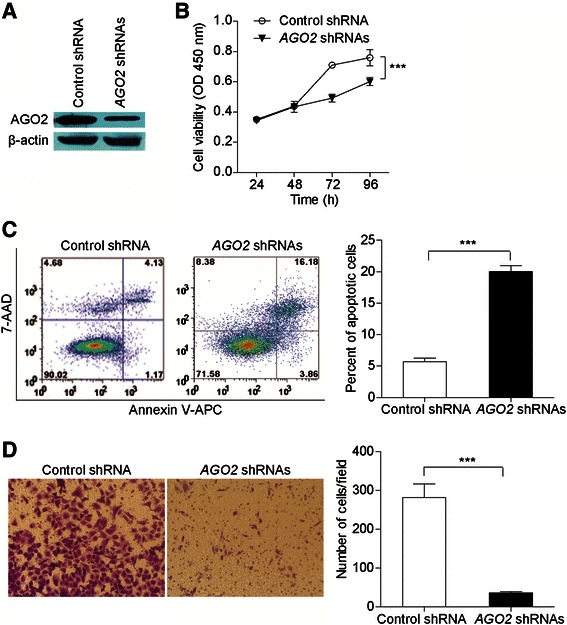
The effect of *AGO2* knockdown on proliferation, apoptosis and migration of NPC cells. **a** Confirmation of *AGO2* knockdown in CNE2Z cells transfected with *AGO2* shRNAs or control shRNA by western blot assay. **b** Cell proliferation was evaluated by CCK-8 assay in CNE2Z cells transfected with *AGO2* shRNAs or control shRNA at different time points (24, 48, 72 and 96 h). The experiments were repeated at least three times, and the points represent the mean values of triplicate tests (mean ± SD). ***P < 0.0001, compared with the controls (ANOVA test). **c** Apoptosis was assessed by flow cytometric analysis of annexin V-APC/7-AAD staining in CNE2Z cells transfected with *AGO2* shRNAs or control shRNA. Representative flow cytometric analysis are shown (*left panel*). The Annexin V-positive cells were regarded as apoptotic cells. The experiments were repeated at least three times, and the histogram (*right panel*) represents the mean values of apoptotic cells from triplicate tests (mean ± SD). ***P < 0.0001, compared with the controls (Unpaired *t* test). **d** Cell migration was evaluated by transwell assay in CNE2Z cells transfected with *AGO2* shRNAs or control shRNA. Representative results are shown (*left panel*). Magnification: 400 ×. The experiments were repeated at least three times, and the histogram (*right panel*) represents the mean numbers of transferred cells from triplicate tests (mean ± SD). ***P < 0.0001, compared with the controls (Unpaired *t* test). CCK-8, Cell Counting Kit-8; SD, standard deviation

Given that the genetic variant of *AGO2* is associated with NPC progression, we speculated that AGO2, which was over-expressed in NPC tissues, might influences the tumorigenic properties of NPC cells. We used three *AGO2* specific shRNAs to knock down *AGO2* in a human nasopharyngeal carcinoma cell line CNE2Z, and western blots assay confirmed that the protein expression level of *AGO2* was remarkably decreased in CNE2Z cells after *AGO2* knockdown (Fig. 2a). The CCK-8 assay was carried out to examine whether *AGO2* knockdown affected NPC cell proliferation. As shown in Fig. 2b, CNE2Z cells transfected with *AGO2* shRNAs had a significant viability reduction over time compared with controls (*P* < 0.0001), suggesting that *AGO2* knockdown significantly inhibited proliferation of NPC cells. Moreover, the percentages of apoptotic cells in CNE2Z cells transfected with *AGO2* shRNAs was increased 14.4 % compared with controls (*P* < 0.0001; Fig. 2c), indicating *AGO2* knockdown induced apoptosis of NPC cells. Furthermore, migratory cells in CNE2Z cells transfected with *AGO2* shRNAs was 36 ± 3, approximately 7.8-fold lower (*P* < 0.0001) than those in CNE2Z cells transfected with control shRNA (282 ± 34), suggesting *AGO2* knockdown reduced migration ability of NPC cells (Fig. 2d).

### Altered gene expression in response to *AGO2* knockdown

To elucidate the comprehensive regulation in transcriptional profile induced by *AGO2*, whole-genome expression microarray analysis was conducted between the CNE2Z cells transfected with *AGO2* shRNAs and control shRNA. A total of 1160 genes were altered significantly, including 767 genes were down-regulated and 393 genes were up-regulated, in CNE2Z cells after *AGO2* knockdown compared with controls (*P* < 0.05; Additional file 2: Table S9).

**Fig. 3:**
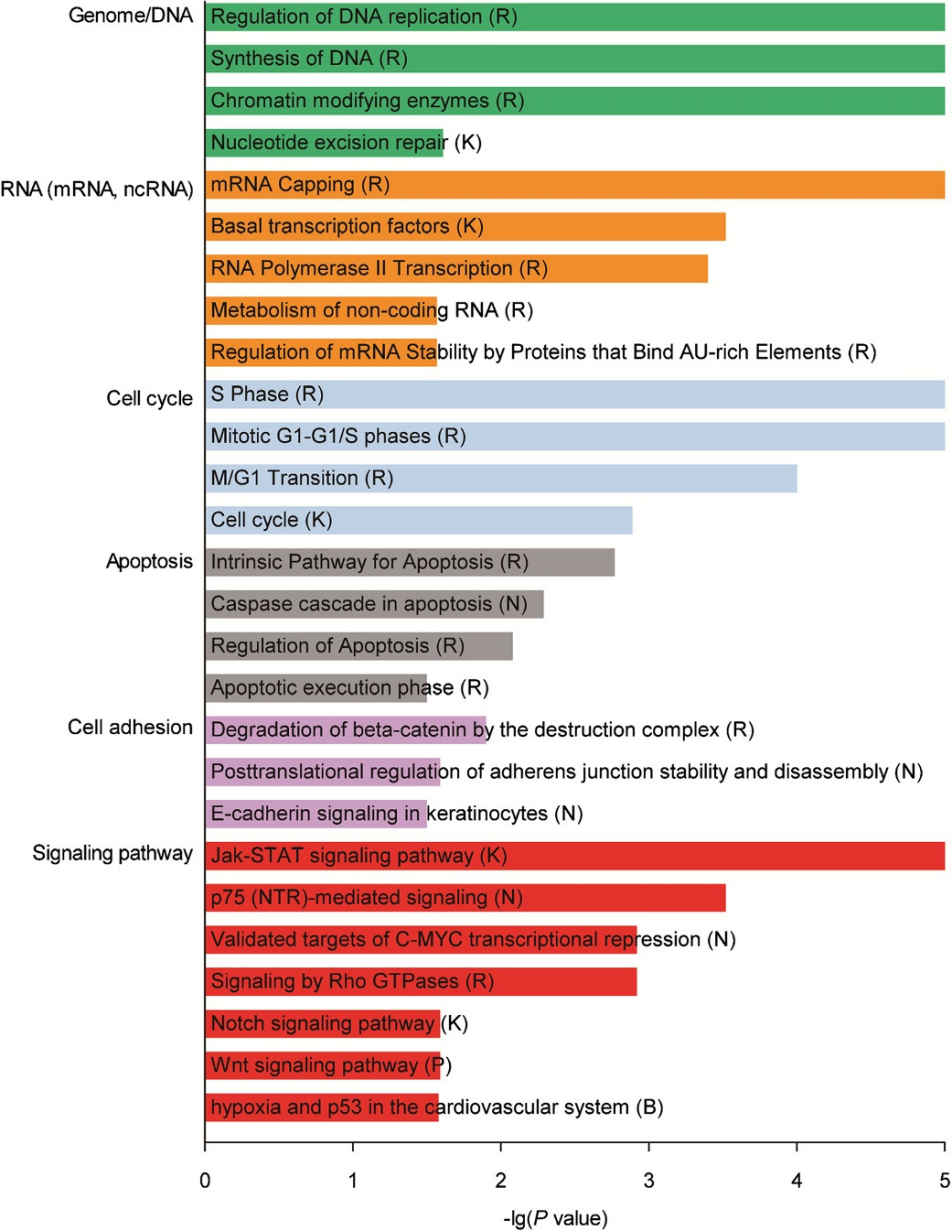
The most enriched biological processes and signaling pathways of the 1160 genes with significantly altered expression following *AGO2* knockdown in CNE2Z cells by cytoscape plug-in of Reactome FI analysis. “G” represents “GO biological process”, “K” represents “KEGG pathway”, “N” represents “NCI PID”, “R” represents “Reactome”, “P” represents “Reactome Pathway” and “B” represents “BioCarta”. The detailed results were shown in Additional file 3: Table S10

To determine whether specific biological processes or signaling pathways were involved in the *AGO2* knockdown-induced anticancer effects, we conducted biological network analysis of these 1160 deferentially expressed genes using the cytoscape plug-in of Reactome FI [23]. Figure 3 shows some of the most significant biological processes or signaling pathways (full list in Additional file 3: Table S10). Remarkably, the majority of the enriched biological processes were related to the cell cycle, apoptosis and cell adhesion, which were hallmarks during the development of cancers. Furthermore, signaling pathways related to the tumor metastasis, namely “Signaling by Rho GTPases” [26], “Notch signaling pathway” [27] and “Wnt signaling pathway” [28] were also significantly enriched. All together, these observations indicate that *AGO2* is associated with the development of NPC by regulating expression of numerous cancer related genes.

## Discussion

In the present study, we systematically evaluated the effect of SNPs in the *AGO2* gene on the risk of occurrence or progression of NPC in two case–control populations of Chinese ancestry. We found that the *AGO2* rs3928672 was significantly associated with the advanced lymph node metastasis of NPC. The functional experiments demonstrated that the genotype of rs3928672 was significantly associated with the expression of AGO2 in NPC tissues, and *AGO2* can play an oncogenic role in the development of NPC by regulating genes related to the tumorigenesis and metastasis. To the best of our knowledge, this is the first report that the genetic polymorphisms of *AGO2* may be risk factor for the progression of NPC, and *AGO2* acts as an oncogene in NPC.

The polymorphisms in the *AGO2* gene have been used to search for susceptibility alleles of a wide spectrum of cancers (Additional file 1: Table S11). For instance, three SNPs, i.e. rs3864659, rs2292779 and rs11786030, in the *AGO2* gene were shown to be associated with breast cancer in Korea population [17, 18]. Unfortunately, on the basis of analysis of 855 NPC cases and 1036 controls in the Guangxi population, we did not find a statistically significant association between these SNPs and the susceptibility to NPC. The inconsistent results may be attributed to different molecular mechanisms of carcinogenesis among cancers, small sample size, marginal statistical significance and different ethnicities of study populations. However, by using the tag SNP approach, we identified a novel tag SNP (i.e. rs3928672) that was associated with the risk of lymph node metastasis of NPC in two Chinese subpopulations. Together, our results support the *AGO2* gene as a susceptibility gene for cancers.

In both study populations, the subjects bearing the rs3928672 A allele (GA + AA genotype) had a significantly increased frequency of involvement of lymph node metastasis of NPC compared with ones bearing GG genotype. Moreover, the rs3928672 A allele carriers having higher AGO2 protein expression than the GG genotype carriers in NPC tissues. Given the role of AGO2 in the development of NPC, one might expect individuals who carry the rs3928672 A allele, and thus have increased expression of AGO2 over a lifetime, may have a higher risk of developing lymph node metastasis of NPC after establishment of this malignancy. However, the rs3928672, which is a polymorphism in intron 4 of *AGO2*, may not have a functional consequence. It is plausible that some other functional polymorphisms in linkage disequilibrium (LD) with the rs3928672 should be responsible for the allele-specific expression. However, the rs3928672 is in low linkage disequilibrium with other SNPs in both the patients and comparison subjects in the Guangxi population as well as in the HapMap CHB (r^2^ < 0.80), indicating that the association of rs3928672 with NPC severity is likely independent. Alternatively, we cannot exclude the possibility that the rs3928672 itself is a functional variant directly affecting the AGO2 production. Introns in eukaryotes fulfill a broad spectrum of functions, such as acting as transposable elements, and are involved in virtually every step of mRNA processing [29]. Indeed, by computer analysis (F-SNP database, http://compbio.cs.queensu.ca/F-SNP) we found that the polymorphism rs3928672 maybe influence binding of transcription factor RUNX1 (runt-related transcription factor 1, also called AML-1) and CAP1 [CAP, adenylate cyclase-associated protein 1 (yeast)], since the sequences flanking rs3928672 A allele (5’-AG*T*GGT-3’) as a potential binding site for the RUNX1, and the sequences flanking G allele (5’-TCACC*G*CT-3’) as a potential binding site for the CAP1, respectively. Therefore, one mechanism by which this could occur is if the risky rs3928672 A allele can influence binding of transcription factor in the intron 4 of *AGO2*. Further studies are needed to clarify which polymorphism(s) may possess functional consequence(s) for AGO2, and in turn to provide mechanistic plausibility for the observed association between rs3928672 and involvement of lymph node metastasis of NPC.

The mechanism of how *AGO2* SNPs regulates human susceptibility to cancer is unknown. However, *AGO2* has significant roles controlling the tumorigenesis and progression of several cancers, including tumor invasion and metastasis [8–10, 14, 15]. This processes appears to be through two different molecular mechanisms, RNAi-dependent gene silencing [8, 30] and RNAi-independent ways such as stabilizing insulator-independent looping [31], facilitating DNA double-strand break repair [32], targeting the intragenic long interspersed nuclear element-1 (LINE-1) transcription complexes [33], and directly regulating downstream gene expression [9]. Consistent with the previous study, we showed that the AGO2 protein was significantly over-expressed in NPC tissues compared with non-cancerous nasopharyngeal epithelium tissues in the present study. Moreover, functional experiments illustrate that *AGO2* knockdown reduced cell proliferation, induced apoptosis, and inhibited migration of NPC cells. These effects of *AGO2* knockdown were further corroborated by genes with altered expression following *AGO2* knockdown, which were functionally clustered in biological processes related with cell cycle, apoptosis and cell migration. Furthermore, several tumorigenesis and metastasis associated genes with altered expression following *AGO2* knockdown were targets of miRNAs (e.g., *TPBG* [34], *JUP* [35], *CDKN1A* [36] and *S100A11* [37] were experimentally validated targets of miR-155 [38, 39]). Therefore, *AGO2* may promote tumorigenesis and metastasis by regulating miRNAs and their targets in NPC. Taken together, our findings indicated that AGO2 over-expression can contribute to NPC malignant behaviors.

In reviewing the results of this study, one must also keep several potential limits in mind. First, as a hospital-based study, our NPC cases were recruited from the hospital, while the controls were selected from the community population; inherent selection bias cannot be completely excluded. However, by further adjustment and stratification in data analyses, the potential confounding effects might have been minimized. Second, a number of association studies have addressed identifying the genes that may relate to the susceptibility to NPC [40–42]. Most of the results, however, could not be replicated in subsequent studies in other populations. Although the highly significant association between the *AGO2* and lymph node metastasis of NPC was strengthened by our two independent case–control studies, our initial findings should be independently verified in other populations with high incidence rate of NPC, such as other Southern Chinese, Singaporeans, and Taiwanese.

## Conclusions

In conclusion, we have shown that the *AGO2* polymorphism may be a genetic risk factor for NPC in Chinese population. If confirmed by other studies, knowledge of genetic factors contributing to the pathogenesis of the NPC as presented here may have implications for the cancer screening and treatment of this disorder.

## Availability of supporting data

Gene expression data have been submitted to Gene Expression Omnibus (GEO) database with the series accession number GSE74154, and these data are available free of charge via the Internet at http://www.ncbi.nlm.nih.gov/geo/query/acc.cgi?acc=GSE74154.

## Additional files

Additional file 1: Table S1. Selected characteristics of patients with NPC and controls in the Guangxi and Guangdong population. **Table S2.** Selected characteristics of patients with NPC and controls for the immunohistochemistry analysis. **Table S3.** Primers used for the genotyping assays. **Table S4.** Genotype frequencies in patients with NPC and controls in the Guangxi population. **Table S5.** Association between AGO2 polymorphisms genotypes and local tumor invasion of NPC. **Table S6.** Association between AGO2 polymorphisms genotypes and lymph node metastasis of NPC. **Table S7.** Risk of lymph node metastasis of NPC associated with the AGO2 rs3928672 by potential risk factors in the Guangxi and Guangdong population. **Table S8.** Correlation between protein expression levels of AGO2 and rs3928672 genotypes in NPC tissues and non-cancerous nasopharyngeal tissues by immunohistochemistry. **Table S11.** Summary of previous studies on the genetic associations between AGO2 and cancers. (DOC 525 kb)
Additional file 2: Table S9. Genes with significantly altered expression following *AGO2* knockdown in CNE2Z cells. (XLS 208 kb)
Additional file 3: Table S10. Biological network analysis of genes with significantly altered expression following *AGO2* knockdown in CNE2Z cells. (XLS 29 kb)

## Abbreviations

AGO2: argonaute 2
CI: confidence interval
IHC: immunohistochemistry
NPC: nasopharyngeal carcinoma
OR: odds ratio
SNP: single nucleotide polymorphism
TNM classification: tumor-node-metastasis classification
