# Supplementary material for: Argonaute 2 and nasopharyngeal carcinoma: a genetic association study and functional analysis
Source: BMC Cancer. 2015 Nov 6;15:862. doi: 10.1186/s12885-015-1895-4 (PMC4636795; doi:10.1186/s12885-015-1895-4)
Supplement: Additional file 1: Table S1. — Selected characteristics of patients with NPC and controls in the Guangxi and Guangdong population. Table S2. Selected characteristics of patients with NPC and controls for the immunohistochemistry analysis. Table S3. Primers used for the genotyping assays. Table S4. Genotype frequencies in patients with NPC and controls in the Guangxi population. Table S5. Association between AGO2 polymorphisms genotypes and local tumor invasion of NPC. Table S6. Association between AGO2 polymorphisms genotypes and lymph node metastasis of NPC. Table S7. Risk of lymph node metastasis of NPC associated with the AGO2 rs3928672 by potential risk factors in the Guangxi and Guangdong population. Table S8. Correlation between protein expression levels of AGO2 and rs3928672 genotypes in NPC tissues and non-cancerous nasopharyngeal tissues by immunohistochemistry. Table S11. Summary of previous studies on the genetic associations between AGO2 and cancers. (DOC 525 kb) [file 12885_2015_1895_MOESM1_ESM.doc]

**Supplementary Tables:**

**Supplementary Table 1.** Selected characteristics of patients with NPC and controls in the Guangxi and Guangdong population

(DOC)

**Supplementary Table 2.** Selected characteristics of patients with NPC and controls for the immunohistochemistry analysis

(DOC)

**Supplementary Table 3.** Primers used for the genotyping assays

(DOC)

**Supplementary Table 4.** Genotype frequencies in patients with NPC and controls in the Guangxi population

(DOC)

**Supplementary Table 5.** Association between *AGO2* polymorphisms genotypes and local tumor invasion of NPC

(DOC)

**Supplementary Table 6.** Association between *AGO2* polymorphisms genotypes and lymph node metastasis of NPC

(DOC)

**Supplementary Table 7.** Risk of lymph node metastasis of NPC associated with the *AGO2* rs3928672 by potential risk factors in the Guangxi and Guangdong population

(DOC)

**Supplementary Table 8**. Correlation between protein expression levels of AGO2 and rs3928672 genotypes in NPC tissues and non-cancerous nasopharyngeal tissues by immunohistochemistry

(DOC)

**Supplementary Table 9.** Genes with significantly altered expression following *AGO2* knockdown in CNE2Z cells

(XLS)

**Supplementary Table 10.** Biological network analysis of genes with significantly altered expression following *AGO2* knockdown in CNE2Z cells

(XLS)

**Supplementary Table 11.** Summary of previous studies on the genetic associations between *AGO2* and cancers

(DOC)

**Supplementary Table 1. Selected characteristics of patients with NPC and controls in the Guangxi and Guangdong population**

| **Characteristics** | **Guangxi population^a^** | | |  |  | **Guangdong population** | | |  |
| --- | --- | --- | --- | --- | --- | --- | --- | --- | --- |
|  | **Cases** | **Controls** | ***P* value** | ***χ^2^*** |  | **Cases** | **Controls** | ***P* value** | ***χ^2^*** |
|  | **(n = 855)** | **(n = 1036)** |  |  |  | **(n = 997)** | **(n = 972)** |  |  |
| Sex, n (%) |  |  |  |  |  |  |  |  |  |
| Male | 616 (72.0) | 749 (72.3) | 0.90 | 0.015 |  | 728 (73.0) | 709 (72.9) | 0.97 | 0.0010 |
| Female | 239 (28.0) | 287 (27.7) |  |  |  | 269 (27.0) | 263 (27.1) |  |  |
| Age, years |  |  |  |  |  |  |  |  |  |
| Mean (SD) | 46.5 (11.9) | 44.6 (12.0) | 0.0011 |  |  | 47.4 (11.1) | 47.4 (11.6) | 0.93 |  |
| ≥ 45, n (%) | 464 (54.3) | 516 (49.8) | 0.053 | 3.74 |  | 583 (58.5) | 578 (59.5) | 0.66 | 0.20 |
| < 45, n (%) | 391 (45.7) | 520 (50.2) |  |  |  | 414 (41.5) | 394 (40.5) |  |  |
| Smoking status, n (%) | |  |  |  |  |  |  |  |  |
| Non-smoker | 598 (69.9) | 719 (69.4) | 0.80 | 0.065 |  | 441 (44.2) | 466 (47.9) | 0.15 | 2.10 |
| Smoker | 257 (30.1) | 317 (30.6) |  |  |  | 546 (54.8) | 506 (52.1) |  |  |
| Unknown | 0 | 0 |  |  |  | 10 (1.0) | 0 |  |  |
| Smoking level, pack-years | |  |  |  |  |  |  |  |  |
| Mean (SD) | 22.9 (12.9) | 24.3 (18.4) | 0.30 |  |  | 26.0 (18.0) | 22.6 (17.8) | 0.096 |  |
| ≥ 24, n (%) | 151 (58.8) | 189 (59.6) | 0.83 | 0.044 |  | 269 (49.3) | 193 (38.1) | 2.48 × 10^-4^ | 13.42 |
| < 24, n (%) | 106 (41.2) | 128 (40.4) |  |  |  | 271 (49.6) | 308 (60.9) |  |  |
| Unknown | 0 | 0 |  |  |  | 6 (1.1) | 5 (1.0) |  |  |
| Drinking status, n (%) |  |  |  |  |  |  |  |  |  |
| Non-drinker | 599 (70.1) | 734 (70.8) | 0.71 | 0.14 |  | 802 (80.4) | 822 (84.6) | 0.11 | 2.60 |
| Drinker | 256 (29.9) | 302 (29.2) |  |  |  | 178 (17.9) | 150 (15.4) |  |  |
| Unknown | 0 | 0 |  |  |  | 17 (1.7) | 0 |  |  |
| Family history^b^, n (%) |  |  |  |  |  |  |  |  |  |
| Negative | 797 (93.2) | 1006 (97.1) | 6.48 × 10^-5^ | 15.96 |  |  |  |  |  |
| Positive | 58 (6.8) | 30 (2.9) |  |  |  |  |  |  |  |
| Nationality, n (%) |  |  |  |  |  |  |  |  |  |
| Han | 628 (73.5) | 904 (87.3) | 2.53 × 10^-14^ | 58.07 |  |  |  |  |  |
| Non-Han^c^ | 227 (26.5) | 132 (12.7) |  |  |  |  |  |  |  |
| Histological type, n (%) | |  |  |  |  |  |  |  |  |
| Poorly differentiated squamous cell carcinoma | 829 (97.0) |  |  |  |  |  |  |  |  |
| Others^d^ | 26 (3.0) |  |  |  |  |  |  |  |  |
| Clinical stage, n (%) |  |  |  |  |  |  |  |  |  |
| I | 41 (4.8) |  |  |  |  | 36 (3.6) |  |  |  |
| II | 395 (46.2) |  |  |  |  | 149 (14.9) |  |  |  |
| III | 259 (30.3) |  |  |  |  | 503 (50.5) |  |  |  |
| IV | 160 (18.7) |  |  |  |  | 253 (25.4) |  |  |  |
| Unknown | 0 |  |  |  |  | 56 (5.6) |  |  |  |
| Local tumor invasion (T classification), n (%) | | | |  |  |  |  |  |  |
| T1 | 170 (19.9) |  |  |  |  | 56 (5.6) |  |  |  |
| T2 | 424 (49.6) |  |  |  |  | 229 (23.0) |  |  |  |
| T3 | 174 (20.4) |  |  |  |  | 482 (48.3) |  |  |  |
| T4 | 87 (10.1) |  |  |  |  | 194 (19.5) |  |  |  |
| Unknown | 0 |  |  |  |  | 36 (3.6) |  |  |  |
| Lymph node involvement (N classification), n (%) | | | |  |  |  |  |  |  |
| N0 | 178 (20.8) |  |  |  |  | 235 (23.6) |  |  |  |
| N1 | 414 (48.4) |  |  |  |  | 384 (38.5) |  |  |  |
| N2 | 185 (21.6) |  |  |  |  | 274 (27.5) |  |  |  |
| N3 | 78 (9.2) |  |  |  |  | 69 (6.9) |  |  |  |
| Unknown | 0 |  |  |  |  | 35 (3.5) |  |  |  |
| Distance metastasis (M classification), n (%) | | | |  |  |  |  |  |  |
| M0 | 835 (97.7) |  |  |  |  | 935 (93.8) |  |  |  |
| M1 | 20 (2.3) |  |  |  |  | 25 (2.5) |  |  |  |
| Unknown | 0 |  |  |  |  | 37 (3.7) |  |  |  |

Abbreviation: SD, standard deviation.

Comparisons of sex, status of smoking and drinking, family history and nationality distributions between patients and controls were performed by use of the *χ^2^* test. Differences of mean age and mean smoking level between patients and controls were analyzed by use of an unpaired *t* test.

^a^Of the Guangxi population (855 cases and 1036 controls) involved in the present study, all were derived from our previous study .

^b^First-degree relatives.

^c^In cases of Guangxi population, non-Han includes Zhuang (n = 211), Dong (n = 1), Hui (n = 1), Miao (n = 1), Mulao (n = 3) and Yao (n = 10) nationality; in controls, non-Han includes Zhuang (n = 132) nationality.

^d^In cases of Guangxi population, other histological types include vesicular nucleus cell carcinoma (n = 14), poorly differentiated adenocarcinoma (n = 4), moderate differentiated squamous cell carcinoma (n = 5) and undifferentiated cancer (n = 3).

**Supplementary Table 2.** Selected characteristics of patients with NPC and controls for the immunohistochemistry analysis

| **Characteristics** | **Cases**  **(n = 37)** | **Controls**  **(n = 18)** |
| --- | --- | --- |
| Sex, n (%) |  |  |
| Male | 21 (56.8) | 14 (77.8) |
| Female | 16 (43.2) | 4 (22.2) |
| Age, years |  |  |
| Mean (SD) | 43.4 (10.0) | 38.6 (12.1) |
| ≥ 45, n (%) | 13 (35.1) | 4 (22.2) |
| < 45, n (%) | 24 (64.9) | 14 (77.8) |
| Smoking status, n (%) |  |  |
| Non-smoker | 26 (70.3) | 15 (83.3) |
| Smoker | 11 (29.7) | 3 (16.7) |
| Smoking level, pack-years |  |  |
| Mean (SD) | 23.8 (12.6) | 27.6 (11.9) |
| ≥ 24, n (%) | 5 (45.5) | 2 (66.7) |
| < 24, n (%) | 6 (54.5) | 1 (33.3) |
| Drinking status, n (%) |  |  |
| Non-drinker | 30 (81.1) | 14 (77.8) |
| Drinker | 7 (18.9) | 4 (22.2) |
| Family history^a^, n (%) |  |  |
| Negative | 37 (100) | 18 (100) |
| Positive | 0 | 0 |
| Nationality, n (%) |  |  |
| Han | 33 (89.2) | 17 (94.4) |
| Non-Han^b^ | 4 (10.8) | 1 (5.6) |
| Histological type, n (%) |  |  |
| Poorly differentiated squamous cell carcinoma | 33 (89.2) |  |
| Others^c^ | 4 (10.8) |  |
| Clinical stage, n (%) |  |  |
| I | 0 |  |
| II | 20 (54.1) |  |
| III | 14 (37.8) |  |
| IV | 3 (8.1) |  |
| Local tumor invasion (T classification), n (%) | |  |
| T1 | 1 (2.7) |  |
| T2 | 25 (67.6) |  |
| T3 | 8 (21.6) |  |
| T4 | 3 (8.1) |  |
| Lymph node involvement (N classification), n (%) | | |
| N0 | 2 (5.4) |  |
| N1 | 24 (64.9) |  |
| N2 | 11 (29.7) |  |
| N3 | 0 |  |
| Distance metastasis (M classification), n (%) | |  |
| M0 | 36 (97.3) |  |
| M1 | 1 (2.7) |  |

Abbreviation: SD, standard deviation.

^a^First-degree relatives.

^b^In cases, non-Han includes Zhuang (n = 4) nationality; in controls, non-Han includes Yao (n = 1) nationality.

^c^In cases, other histological types include vesicular nucleus cell carcinoma (n = 1), undifferentiated cancer (n = 1), carcinoma in situ (n = 1) and moderate differentiated squamous cell carcinoma (n = 1). The histological type of all tissues in the controls was chronic inflammation.

**Supplementary Table 3.** Primers used for the genotyping assays

| **SNPs** | **Primers** | **Sequences** |
| --- | --- | --- |
| rs4961280 | Forward | 5’-CAGATGCCCACCCTCTCT-3’ |
|  | Reverse | 5’-AGGGCAGTTGGGCCTCAG-3’ |
|  | Extension | 5’-AGAGCGAGTGACGCATACTACTGCCCCTGTCTCCTTCACATATCC-3’ |
| rs11996715 | Forward | 5’-ATCCCTTAAACTGCACTTTTACTTT-3’ |
|  | Reverse | 5’-ATCTGCGAACTGGGAAGATT-3’ |
|  | Extension | 5’-GCGGTAGGTTCCCGACATATATTCATGATTATTTAAAAATTTCC-3’ |
| rs4961226 | Forward | 5’-CATACCTTCATGGGGACCAC-3’ |
|  | Reverse | 5’-GTGTTACACCCCTCGCAGAT-3’ |
|  | Extension | 5’-ACGTAAGACCACTCAAGACCAATGGCTCTATAAACCACAGTGAAG-3’ |
| rs10088596 | Forward | 5’-AACCTCAATGACACCACACTC-3’ |
|  | Reverse | 5’-TCAGATTCAATGCGTTTTTAAA-3’ |
|  | Extension | 5’-CACCGCTATCAACAGACTTGTTTAAAATGTTCCAGCCTGTTCATC-3’ |
| rs883596 | Forward | 5’-CTCGTATCCAGGGGGTGC-3’ |
|  | Reverse | 5’-TCATGGTGTGTGATTGAGGA-3’ |
|  | Extension | 5’-AGATAGAGTCGATGCCAGCTAGAGGGCTTGGGGGCAGCATCAAAA-3’ |
| rs13261055 | Forward | 5’-TAATATTCACGGGCAACACC-3’ |
|  | Reverse | 5’-TTTTATTTCTCTTGTGTCGTTGC-3’ |
|  | Extension | 5’-CACCGCTATCAACAGACTTGGAACAGCTCCCTAACAGGCACTGTA-3’ |
| rs7001653 | Forward | 5’-TGCTCTTCTTGGGTGGTTTT-3’ |
|  | Reverse | 5’-CCCGGACCACACTTAGAGAA-3’ |
|  | Extension | 5’-CAGTCAACAATCCAGATCAAATGTTCCACTTTAATTAAATTGTAT-3’ |
| rs7819727 | Forward | 5’-AAACCAGGCCCCTCCACC-3’ |
|  | Reverse | 5’-GGAAGGCTTCTTAGGAGAAATAG-3’ |
|  | Extension | 5’-CAACAAGTAATCCGCAGACTAGAGTCCAGGCACAGACGCAGCTCG-3’ |
| rs7009635 | Forward | 5’-TGTCACTATACAGACCTGGAAACTAA-3’ |
|  | Reverse | 5’-TAGAAAGTTGCCTCATACCCTC-3’ |
|  | Extension | 5’-CAGAATAGCCACGCCTAGATGACACACATCAGAATGCCGTGACTT-3’ |
| rs7005286 | Forward | 5’-AAATAAATGGTGTTCCCATGC-3’ |
|  | Reverse | 5’-AAACACACAAAAAAACACGC-3’ |
|  | Extension | 5’-ACGTAAGACCACTCAAGACCCCCGTCAGCTCCGCTGAGTGTTTCC-3’ |
| rs11776034 | Forward | 5’-TTTAGTAAAGAAAGCTGCTTAACCC-3’ |
|  | Reverse | 5’-ATATCTGTAAAACTAAACAGTTGGATTCA-3’ |
|  | Extension | 5’-ACGCACGTCCACGGTGATTTTATTTAAAGAACCTCATAGAACTCT-3’ |
| rs3735805 | Forward | 5’-AGGTGGTCCACACCCGAG-3’ |
|  | Reverse | 5’-AGGGCTGGGAGTGACCTT-3’ |
|  | Extension | 5’-ACCGCACTAAGCAATGTATCACTGCCGCCTCTCAACAGCCCAAAG-3’ |
| rs2977490 | Forward | 5’-ATGTGACAGGTTACAGATGGTATATATG-3’ |
|  | Reverse | 5’-AAGTGAGCCAATACTGCACC-3’ |
|  | Extension | 5’-CTCACTATCTGACAAGCCACCATTCGCCACTTTTGTTGAGACAGG-3’ |
| rs2292773 | Forward | 5’-ATCCAAGAATGAGCCGTTC-3’ |
|  | Reverse | 5’-TCATGAGGCACTTGCCAT-3’ |
|  | Extension | 5’-CAGCCATCCATTCACTATCTCGGACAGATTTCAGACCCTGCGGGG-3’ |
| rs3928672 | Forward | 5’-ATCTCTTGCCCTTGGGAC-3’ |
|  | Reverse | 5’-ATTTGTGGTAAAAGAGTAATCCTGAG-3’ |
|  | Extension | 5’-CAAGACCGCAACTAGATACAGTCTGCAACTCTAGGGACCATCACC-3’ |
| rs2271735 | Forward | 5’-AATGTAGCCATCAGATGAGGTAG-3’ |
|  | Reverse | 5’-TGCTTCTAGTCCTGAGATCATG-3’ |
|  | Extension | 5’-GGCTATGATTCGCAATGCTTTCCATTTCCCAGAGCGGGAGTGGGG-3’ |
| rs2977481 | Forward | 5’-TAGTTGATGGGTGGGTGG-3’ |
|  | Reverse | 5’-CACCCACTTTATCCACCCA-3’ |
|  | Extension | 5’-GTGATTCTGTACGTGTCGCCCCCATCTTATCCACCGATCATCAAC-3’ |
| rs2292779 | Forward | 5’-GCATCGAGATCAAGGTGTGG-3’ |
|  | Reverse | 5’-AGAGTCAGCCCTGCAAGTG-3’ |
|  | Extension | 5’-CTCACTATCTGACAAGCCACACGGAAGTCCATCTGAAGTAAGCCC-3’ |
| rs2944764 | Forward | 5’-TTACTTCTCCATGCTCACTGC-3’ |
|  | Reverse | 5’-TTTCCCTGTCACGTACCATT-3’ |
|  | Extension | 5’-ACAGATCACTCACCGACTAAGGGTCCCAGGAGTGAGAGGGGCACC-3’ |
| rs11166983 | Forward | 5’-TTTAAATGCCTCACCTAGGG-3’ |
|  | Reverse | 5’-GTAAGTGGCCTTCCTAAGCAG-3’ |
|  | Extension | 5’-ACAAGAACTCCATGACTCAACAGGGAGCCAAGTCCTTTCTTTTAG-3’ |
| rs12542354 | Forward | 5’-TACGATGGGCTCATGACC-3’ |
|  | Reverse | 5’-TATTTATTGAGTACCTACTGTGTGCAG-3’ |
|  | Extension | 5’-GCAACATAAGACCGCTCAACGGTGCCTCCACCAGGACTTCCGGCC-3’ |
| rs13252337 | Forward | 5’-CAGGTTCTCTTGTGAGTGTG-3’ |
|  | Reverse | 5’-AAGAACGAGCGGGTGAGT-3’ |
|  | Extension | 5’-CTAACTAAGCTACGCCGACACTCGGGGTGTGGGGCTGAGGGGAGA-3’ |
| rs2977469 | Forward | 5’-ATCTTAAAACCCTCCAAAACAAC-3’ |
|  | Reverse | 5’-AAGAGAACCTGCTGGCCA-3’ |
|  | Extension | 5’-AAGTACCACGTCAACGTCACCAGGCTCTAGGGTGACTCTGTCCTC-3’ |
| rs2977464 | Forward | 5’-TGACTGCGCCAAGTGCTC-3’ |
|  | Reverse | 5’-ATGGGGTGGAAGCTGGAA-3’ |
|  | Extension | 5’-CAGCCATCCATTCACTATCTGAATCGACGCCAAGTCTGCCCCATG-3’ |
| rs2977462 | Forward | 5’-CTGGGCAACAGAGAGAGACT-3’ |
|  | Reverse | 5’-AGCCTGCTTTATATCTTTGATATGAT-3’ |
|  | Extension | 5’-CAACAATACGAGCCAGCAAGAAGCATCATAACTACATATAGTAAA-3’ |

**Supplementary Table 4.** Genotype frequencies in patients with NPC and controls in the Guangxi population

| **SNPs** | **Populations^a^** | **Genotypes** | **Cases (n = 855)** | **Controls (n = 1036)** | **Model^b^** | **OR (95% CI)^c^** | ***P* value^d^** |
| --- | --- | --- | --- | --- | --- | --- | --- |
| rs4961280 | GX | CC/CA/AA | 601/185/15 | 768/237/17 | Dominant | 0.99 (0.79-1.23) | 0.91 |
| rs11996715 | GX | TT/TG/GG | 309/341/95 | 420/464/123 | Log-additive | 1.02 (0.89-1.18) | 0.77 |
| rs4961226 | GX | GG/TG/TT | 343/344/93 | 430/472/123 | Dominant | 0.92 (0.76-1.11) | 0.37 |
| rs10088596 | GX | CC/TC/TT | 225/406/195 | 270/534/226 | Recessive | 1.10 (0.88-1.38) | 0.40 |
| rs13261055 | GX | AA/GA/GG | 331/382/109 | 422/483/116 | Recessive | 1.25 (0.94-1.66) | 0.13 |
| rs7001653 | GX | CC/TC/TT | 251/370/204 | 313/483/220 | Recessive | 1.20 (0.96-1.51) | 0.10 |
| rs7819727 | GX | GG/GA/AA | 302/391/142 | 350/513/169 | Dominant | 0.90 (0.74-1.09) | 0.29 |
| rs7009635 | GX | TT/TC/CC | 373/377/97 | 422/498/111 | Dominant | 0.90 (0.74-1.09) | 0.27 |
| rs7005286 | GX | GG/GA/AA | 438/321/67 | 587/378/59 | Log-additive | 1.17 (1.01-1.36) | 0.042 |
| rs11776034 | GX | CC/CG/GG | 399/328/77 | 480/462/82 | Recessive | 1.28 (0.92-1.79) | 0.15 |
| rs3735805 | GX | CC/TC/TT | 434/341/71 | 572/392/67 | Log-additive | 1.17 (1.01-1.35) | 0.042 |
| rs2292773 | GX | AA/GA/GG | 424/352/51 | 552/407/66 | Dominant | 1.09 (0.90-1.31) | 0.37 |
| rs3928672 | GX | GG/GA/AA | 395/365/81 | 543/404/84 | Dominant | 1.24 (1.03-1.49) | 0.024 |
| rs2271735 | GX | CC/CA/AA | 472/304/49 | 574/381/66 | Recessive | 0.92 (0.62-1.36) | 0.67 |
| rs2292779 | GX | GG/CG/CC | 377/359/60 | 469/464/94 | Recessive | 0.81 (0.58-1.14) | 0.22 |
| rs2944764 | GX | GG/GA/AA | 322/390/98 | 413/476/139 | Recessive | 0.89 (0.67-1.18) | 0.40 |
| rs11166983 | GX | CC/TC/TT | 303/417/132 | 345/520/168 | Log-additive | 0.95 (0.83-1.09) | 0.48 |
| rs12542354 | GX | GG/GA/AA | 464/316/63 | 649/325/51 | Log-additive | 1.32 (1.13-1.54) | 0.00040^d^ |
|  | GD | GG/GA/AA | 576/331/63 | 538/352/55 | Log-additive | 0.95 (0.82-1.10) | 0.49 |
| rs13252337 | GX | AA/GA/GG | 426/361/53 | 521/423/81 | Recessive | 0.79 (0.55-1.14) | 0.21 |
| rs2977469 | GX | CC/TC/TT | 268/421/146 | 318/525/186 | Log-additive | 0.93 (0.82-1.07) | 0.33 |
| rs2977464 | GX | CC/TC/TT | 403/380/60 | 556/408/67 | Dominant | 1.30 (1.08-1.57) | 0.0052 |
| rs2977462 | GX | AA/GA/GG | 297/395/128 | 333/507/167 | Dominant | 0.84 (0.69-1.03) | 0.088 |

Abbreviation: OR, odds ratio; CI, confidence interval.

Note: The number of genotyped samples varies due to genotyping failure.

^a^GX, Guangxi population; GD, Guangdong population.

^b^The model with the smallest Akaike’s information criterion (AIC) value was defined as the best model for each SNP.

^c^In the Guangxi population, the ORs, 95% CIs and *P* values were adjusted for sex, age, status of smoking and drinking, smoking level, family history and nationality. In the Guangdong population, the ORs, 95% CIs and *P* values were adjusted for sex, age, status of smoking and drinking, and smoking level.

^d^A *P* value of 0.0036 (0.05/14.01, correction factor: 14.01) was used as the criterion of statistical significance in Guangxi population.

**Supplementary Table 5.** Association between *AGO2* polymorphisms genotypes and local tumor invasion of NPC

| **SNPs** | **Populations^a^** | **Genotypes** | **T classification, n** | | | | **T2 + T3 + T4 vs. T1** | |  |  | **T3 + T4 vs. T1 + T2** | |  |  | **T4 vs. T1 + T2 + T3** | |  |
| --- | --- | --- | --- | --- | --- | --- | --- | --- | --- | --- | --- | --- | --- | --- | --- | --- | --- |
|  |  |  | **T1** | **T2** | **T3** | **T4** | **Model^b^** | **OR (95% CI)^c^** | ***P*^c^** |  | **Model^b^** | **OR (95% CI)^c^** | ***P*^c^** |  | **Model^b^** | **OR (95% CI)^c^** | ***P*^c^** |
| rs4961280 | GX | CC/CA/AA | 121/38/6 | 299/88/6 | 118/40/2 | 63/19/1 | Recessive | 0.41 (0.14-1.21) | 0.12 |  | Recessive | 0.68 (0.19-2.48) | 0.55 |  | Recessive | 0.72 (0.09-5.66) | 0.74 |
| rs11996715 | GX | TT/TG/GG | 76/59/18 | 146/177/54 | 59/64/16 | 28/41/7 | Dominant | 1.54 (1.07-2.21) | 0.021 |  | Recessive | 0.79 (0.48-1.32) | 0.36 |  | Recessive | 0.67 (0.29-1.53) | 0.33 |
| rs4961226 | GX | GG/TG/TT | 73/64/25 | 155/190/41 | 72/59/21 | 43/31/6 | Recessive | 0.68 (0.41-1.13) | 0.15 |  | Dominant | 0.75 (0.55-1.02) | 0.070 |  | Log-additive | 0.73 (0.50-1.05) | 0.085 |
| rs10088596 | GX | CC/TC/TT | 48/82/37 | 105/212/93 | 49/71/46 | 23/41/19 | Log-additive | 1.11 (0.87-1.41) | 0.41 |  | Recessive | 1.25 (0.88-1.77) | 0.22 |  | Dominant | 1.00 (0.60-1.68) | 0.99 |
| rs13261055 | GX | AA/GA/GG | 65/83/19 | 157/188/60 | 77/70/18 | 32/41/12 | Log-additive | 1.00 (0.77-1.29) | 0.99 |  | Dominant | 0.80 (0.59-1.09) | 0.16 |  | Log-additive | 1.07 (0.76-1.50) | 0.70 |
| rs7001653 | GX | CC/TC/TT | 53/80/34 | 124/182/104 | 52/69/44 | 22/39/22 | Recessive | 1.34 (0.88-2.05) | 0.17 |  | Recessive | 1.16 (0.82-1.65) | 0.40 |  | Dominant | 1.18 (0.70-1.98) | 0.54 |
| rs7819727 | GX | GG/GA/AA | 58/83/27 | 148/201/64 | 60/70/37 | 36/37/14 | Dominant | 0.92 (0.64-1.31) | 0.63 |  | Recessive | 1.38 (0.93-2.03) | 0.11 |  | Dominant | 0.77 (0.49-1.22) | 0.26 |
| rs7009635 | GX | TT/TC/CC | 72/73/23 | 190/186/44 | 73/81/19 | 38/37/11 | Recessive | 0.82 (0.49-1.36) | 0.44 |  | Dominant | 1.06 (0.79-1.44) | 0.70 |  | Recessive | 1.21 (0.61-2.39) | 0.60 |
| rs7005286 | GX | GG/GA/AA | 100/52/14 | 203/171/34 | 90/65/13 | 45/33/6 | Dominant | 1.46 (1.03-2.08) | 0.034 |  | Recessive | 0.86 (0.49-1.51) | 0.59 |  | Recessive | 0.80 (0.33-1.94) | 0.61 |
| rs11776034 | GX | CC/CG/GG | 76/70/18 | 197/163/36 | 81/66/15 | 45/29/8 | Log-additive | 0.86 (0.66-1.11) | 0.25 |  | Dominant | 0.88 (0.64-1.19) | 0.40 |  | Dominant | 0.75 (0.47-1.20) | 0.22 |
| rs3735805 | GX | CC/TC/TT | 93/62/14 | 207/177/36 | 90/69/13 | 44/33/8 | Dominant | 1.23 (0.87-1.74) | 0.23 |  | Dominant | 0.92 (0.68-1.24) | 0.58 |  | Recessive | 1.19 (0.54-2.60) | 0.67 |
| rs2292773 | GX | AA/GA/GG | 85/69/12 | 219/168/24 | 79/78/9 | 41/37/6 | Recessive | 0.76 (0.38-1.51) | 0.44 |  | Dominant | 1.16 (0.85-1.57) | 0.35 |  | Log-additive | 1.07 (0.73-1.55) | 0.73 |
| rs3928672 | GX | GG/GA/AA | 76/71/21 | 203/175/36 | 80/77/15 | 36/42/9 | Recessive | 0.67 (0.39-1.15) | 0.16 |  | Dominant | 1.13 (0.84-1.53) | 0.41 |  | Dominant | 1.29 (0.82-2.04) | 0.27 |
| rs2271735 | GX | CC/CA/AA | 80/70/16 | 245/142/20 | 101/57/8 | 46/35/5 | Log-additive | 0.66 (0.50-0.87) | 0.0031^d^ |  | Recessive | 0.83 (0.43-1.62) | 0.58 |  | Dominant | 1.21 (0.77-1.91) | 0.40 |
|  | GD | CC/CA/AA | 32/18/5 | 119/82/5 | 250/178/46 | 98/86/7 | Log-additive | 1.12 (0.72-1.75) | 0.61 |  | Recessive | 0.78 (0.47-1.30) | 0.34 |  | Dominant | 1.10 (0.79-1.52) | 0.57 |
| rs2292779 | GX | GG/CG/CC | 77/71/12 | 184/183/27 | 78/67/12 | 38/38/7 | Recessive | 0.81 (0.43-1.53) | 0.52 |  | Dominant | 0.98 (0.72-1.33) | 0.88 |  | Dominant | 1.15 (0.72-1.83) | 0.56 |
| rs2944764 | GX | GG/GA/AA | 58/83/24 | 169/187/47 | 63/80/18 | 32/40/9 | Log-additive | 0.81 (0.63-1.05) | 0.12 |  | Recessive | 0.91 (0.56-1.47) | 0.70 |  | Log-additive | 0.97 (0.68-1.38) | 0.84 |
| rs11166983 | GX | CC/TC/TT | 57/79/33 | 160/204/60 | 56/89/28 | 30/45/11 | Recessive | 0.68 (0.44-1.06) | 0.098 |  | Dominant | 1.11 (0.81-1.52) | 0.51 |  | Recessive | 0.77 (0.39-1.51) | 0.44 |
| rs12542354 | GX | GG/GA/AA | 104/51/14 | 220/169/29 | 90/68/12 | 50/28/8 | Dominant | 1.43 (1.01-2.04) | 0.045 |  | Dominant | 1.03 (0.76-1.40) | 0.84 |  | Recessive | 1.35 (0.61-2.98) | 0.47 |
| rs13252337 | GX | AA/GA/GG | 71/80/17 | 227/166/23 | 87/76/7 | 41/39/6 | Log-additive | 0.69 (0.52-0.90) | 0.0073 |  | Recessive | 0.73 (0.38-1.42) | 0.35 |  | Dominant | 1.17 (0.74-1.84) | 0.49 |
| rs2977469 | GX | CC/TC/TT | 71/73/25 | 123/217/74 | 46/89/33 | 28/42/14 | Dominant | 1.68 (1.18-2.40) | 0.0044 |  | Dominant | 1.15 (0.83-1.59) | 0.41 |  | Log-additive | 0.89 (0.64-1.24) | 0.49 |
| rs2977464 | GX | CC/TC/TT | 94/59/16 | 189/207/26 | 77/81/11 | 43/33/7 | Dominant | 1.52 (1.08-2.16) | 0.016 |  | Dominant | 1.01 (0.75-1.37) | 0.93 |  | Dominant | 0.83 (0.53-1.32) | 0.44 |
| rs2977462 | GX | AA/GA/GG | 66/77/25 | 151/189/64 | 57/82/27 | 23/47/12 | Dominant | 1.18 (0.82-1.68) | 0.37 |  | Dominant | 1.31 (0.95-1.81) | 0.098 |  | Dominant | 1.57 (0.94-2.62) | 0.080 |

Abbreviation: T, local tumor invasion; OR, odds ratio; CI, confidence interval; NA, not available.

Note: The number of genotyped samples varies due to genotyping failure.

^a^GX, Guangxi population; GD, Guangdong population.

^b^The model with the smallest Akaike’s information criterion (AIC) value was defined as the best model for each SNP.

^c^In the Guangxi population, the ORs, 95% CIs and *P* values were adjusted for sex, age, status of smoking and drinking, smoking level, family history and nationality. In the Guangdong population, the ORs, 95% CIs and *P* values were adjusted for sex, age, status of smoking and drinking, and smoking level.

^d^A *P* value of 0.0036 (0.05/14.01, correction factor: 14.01) was used as the criterion of statistical significance in Guangxi population.

**Supplementary Table 6. Association between *AGO2* polymorphisms genotypes and lymph node metastasis of NPC**

| **SNPs** |  | **Populations^a^** | **Genotypes** | **N classification, n** | | | | **N1 + N2 + N3 vs. N0** | |  |  | **N2 + N3 vs. N0 + N1** | |  |  | **N3 vs. N0 + N1 + N2** | |  |
| --- | --- | --- | --- | --- | --- | --- | --- | --- | --- | --- | --- | --- | --- | --- | --- | --- | --- | --- |
|  |  |  |  | **N0** | **N1** | **N2** | **N3** | **Model^b^** | **OR (95% CI)^c^** | ***P*^c^** |  | **Model^b^** | **OR (95% CI)^c^** | ***P*^c^** |  | **Model^b^** | **OR (95% CI)^c^** | ***P*^c^** |
| rs4961280 |  | GX | CC/CA/AA | 123/38/5 | 291/90/5 | 134/39/4 | 53/18/1 | Recessive | 0.58 (0.19-1.74) | 0.35 |  | Recessive | 1.19 (0.39-3.57) | 0.76 |  | Dominant | 1.12 (0.64-1.94) | 0.70 |
| rs11996715 |  | GX | TT/TG/GG | 58/79/20 | 148/165/45 | 71/69/21 | 32/28/9 | Dominant | 0.79 (0.55-1.14) | 0.20 |  | Dominant | 0.81 (0.59-1.12) | 0.20 |  | Dominant | 0.79 (0.48-1.31) | 0.36 |
| rs4961226 |  | GX | GG/TG/TT | 84/69/13 | 152/175/48 | 72/72/25 | 35/28/7 | Log-additive | 1.38 (1.05-1.80) | 0.018 |  | Recessive | 1.21 (0.76-1.92) | 0.43 |  | Dominant | 0.80 (0.49-1.32) | 0.38 |
| rs10088596 |  | GX | CC/TC/TT | 51/89/32 | 112/190/99 | 42/87/47 | 20/40/17 | Recessive | 1.45 (0.95-2.22) | 0.082 |  | Dominant | 1.25 (0.89-1.77) | 0.19 |  | Dominant | 1.08 (0.63-1.85) | 0.78 |
| rs13261055 |  | GX | AA/GA/GG | 72/79/21 | 160/183/52 | 71/80/26 | 28/40/10 | Log-additive | 1.07 (0.83-1.38) | 0.58 |  | Recessive | 1.09 (0.71-1.69) | 0.69 |  | Dominant | 1.19 (0.73-1.94) | 0.48 |
| rs7001653 |  | GX | CC/TC/TT | 50/79/42 | 133/173/97 | 48/83/45 | 20/35/20 | Dominant | 0.93 (0.64-1.35) | 0.70 |  | Dominant | 1.25 (0.89-1.74) | 0.19 |  | Dominant | 1.21 (0.70-2.07) | 0.49 |
| rs7819727 |  | GX | GG/GA/AA | 67/86/21 | 139/184/79 | 64/87/31 | 32/34/11 | Recessive | 1.66 (1.01-2.74) | 0.039 |  | Dominant | 0.94 (0.69-1.28) | 0.71 |  | Dominant | 0.77 (0.47-1.24) | 0.28 |
| rs7009635 |  | GX | TT/TC/CC | 73/88/16 | 183/173/51 | 84/79/22 | 33/37/8 | Recessive | 1.37 (0.78-2.43) | 0.26 |  | Dominant | 0.95 (0.71-1.28) | 0.74 |  | Recessive | 0.88 (0.41-1.90) | 0.75 |
| rs7005286 |  | GX | GG/GA/AA | 100/63/9 | 198/165/34 | 90/75/16 | 50/18/8 | Log-additive | 1.27 (0.96-1.67) | 0.087 |  | Dominant | 0.95 (0.71-1.28) | 0.74 |  | Dominant | 0.56 (0.34-0.93) | 0.021 |
| rs11776034 |  | GX | CC/CG/GG | 85/69/15 | 187/164/38 | 93/65/17 | 34/30/7 | Log-additive | 1.06 (0.81-1.37) | 0.69 |  | Dominant | 0.89 (0.65-1.20) | 0.44 |  | Dominant | 1.05 (0.64-1.72) | 0.84 |
| rs3735805 |  | GX | CC/TC/TT | 107/55/14 | 202/178/30 | 85/80/17 | 40/28/10 | Dominant | 1.61 (1.14-2.26) | 0.0061 |  | Log-additive | 1.20 (0.95-1.50) | 0.12 |  | Recessive | 1.73 (0.84-3.55) | 0.16 |
| rs2292773 |  | GX | AA/GA/GG | 91/75/6 | 205/171/23 | 96/67/16 | 32/39/6 | Recessive | 2.02 (0.84-4.85) | 0.090 |  | Recessive | 1.78 (0.99-3.20) | 0.059 |  | Dominant | 1.58 (0.98-2.56) | 0.061 |
| rs3928672 |  | GX | GG/GA/AA | 91/72/14 | 187/177/42 | 95/69/16 | 22/47/9 | Log-additive | 1.22 (0.94-1.59) | 0.13 |  | Dominant | 1.11 (0.83-1.50) | 0.47 |  | Dominant | 2.47 (1.47-4.13) | 0.00030^d^ |
|  |  | GD | GG/GA/AA | 123/87/16 | 184/152/36 | 134/105/21 | 25/37/3 | Log-additive | 1.20 (0.94-1.52) | 0.13 |  | Dominant | 1.13 (0.86-1.48) | 0.38 |  | Dominant | 1.75 (1.03-2.98) | 0.034 |
| rs2271735 |  | GX | CC/CA/AA | 106/62/6 | 229/147/22 | 104/63/12 | 33/32/9 | Recessive | 2.03 (0.84-4.87) | 0.087 |  | Recessive | 1.88 (1.03-3.41) | 0.043 |  | Log-additive | 1.74 (1.20-2.53) | 0.0040 |
| rs2292779 |  | GX | GG/CG/CC | 75/78/13 | 173/181/30 | 94/65/14 | 35/35/3 | Dominant | 0.93 (0.65-1.31) | 0.66 |  | Dominant | 0.77 (0.57-1.04) | 0.089 |  | Recessive | 0.47 (0.14-1.55) | 0.17 |
| rs2944764 |  | GX | GG/GA/AA | 69/83/13 | 157/184/52 | 74/80/23 | 22/43/10 | Recessive | 1.84 (0.99-3.40) | 0.040 |  | Recessive | 1.20 (0.76-1.90) | 0.43 |  | Dominant | 1.62 (0.96-2.74) | 0.062 |
| rs11166983 |  | GX | CC/TC/TT | 71/87/20 | 143/205/64 | 68/82/34 | 21/43/14 | Recessive | 1.57 (0.95-2.62) | 0.070 |  | Recessive | 1.39 (0.94-2.07) | 0.10 |  | Dominant | 1.51 (0.89-2.55) | 0.12 |
| rs12542354 |  | GX | GG/GA/AA | 103/67/7 | 207/161/39 | 102/65/15 | 52/23/2 | Recessive | 2.35 (1.05-5.28) | 0.023 |  | Dominant | 0.77 (0.57-1.04) | 0.090 |  | Log-additive | 0.58 (0.38-0.90) | 0.0099 |
| rs13252337 |  | GX | AA/GA/GG | 98/66/10 | 198/189/21 | 100/67/13 | 30/39/9 | Dominant | 1.33 (0.95-1.86) | 0.098 |  | Recessive | 1.77 (0.99-3.16) | 0.058 |  | Log-additive | 1.66 (1.15-2.40) | 0.0076 |
| rs2977469 |  | GX | CC/TC/TT | 41/92/41 | 136/216/51 | 61/81/39 | 30/32/15 | Log-additive | 0.68 (0.53-0.86) | 0.0018 ^d^ |  | Recessive | 1.40 (0.96-2.05) | 0.085 |  | Dominant | 0.69 (0.42-1.12) | 0.14 |
|  |  | GD | CC/TC/TT | 96/105/34 | 156/177/51 | 117/123/34 | 28/32/8 | Log-additive | 0.78 (0.52-1.15) | 0.21 |  | Recessive | 1.14 (0.75-1.75) | 0.54 |  | Dominant | 1.11 (0.80-1.54) | 0.54 |
| rs2977464 |  | GX | CC/TC/TT | 96/74/7 | 180/198/30 | 83/76/21 | 44/32/2 | Log-additive | 1.36 (1.03-1.81) | 0.029 |  | Recessive | 1.41 (0.81-2.45) | 0.23 |  | Log-additive | 0.65 (0.43-0.98) | 0.036 |
| rs2977462 |  | GX | AA/GA/GG | 53/91/29 | 154/184/58 | 64/84/29 | 26/36/12 | Dominant | 0.74 (0.51-1.06) | 0.097 |  | Log-additive | 1.04 (0.84-1.29) | 0.72 |  | Dominant | 1.07 (0.65-1.78) | 0.79 |

Abbreviation: N, lymph node involvement; OR, odds ratio; CI, confidence interval.

Note: The number of genotyped samples varies due to genotyping failure.

^a^GX, Guangxi population; GD, Guangdong population.

^b^The model with the smallest Akaike’s information criterion (AIC) value was defined as the best model for each SNP.

^c^In the Guangxi population, the ORs, 95% CIs and *P* values were adjusted for sex, age, status of smoking and drinking, smoking level, family history and nationality. In the Guangdong population, the ORs, 95% CIs and *P* values were adjusted for sex, age, status of smoking and drinking, and smoking level.

^d^A *P* value of 0.0036 (0.05/14.01, correction factor: 14.01) was used as the criterion of statistical significance in Guangxi population.

**Supplementary Table 7.** Risk of lymph node metastasis of NPC associated with the *AGO2* rs3928672 by potential risk factors in the Guangxi and Guangdong population

| **Categories** | **Guangxi population** | | | |  | **Guangdong population** | | | |
| --- | --- | --- | --- | --- | --- | --- | --- | --- | --- |
|  | **GG^a^** | **GA + AA^a^** | **OR (95% CI)^b^** | ***P*_homogeneity_^c^** |  | **GG^a^** | **GA + AA^a^** | **OR (95% CI)^b^** | ***P*_homogeneity_^c^** |
| Sex |  |  |  |  |  |  |  |  |  |
| Male | 16/265 | 46/279 | 2.78 (1.53-5.05) | 0.40 |  | 21/310 | 37/302 | 1.98 (1.12-3.49) | 0.24 |
| Female | 6/108 | 10/111 | 1.65 (0.58-4.70) |  |  | 4/124 | 3/111 | 0.75 (0.16-3.47) |  |
| Age, years |  |  |  |  |  |  |  |  |  |
| ≥ 45 | 11/199 | 29/216 | 2.44 (1.18-5.04) | 0.95 |  | 15/242 | 22/255 | 1.28 (0.64-2.56) | 0.20 |
| < 45 | 11/174 | 27/174 | 2.52 (1.21-5.26) |  |  | 10/192 | 18/158 | 2.57 (1.13-5.83) |  |
| Smoking status |  |  |  |  |  |  |  |  |  |
| Nonsmoker | 14/261 | 31/283 | 2.04 (1.06-3.92) | 0.37 |  | 5/201 | 6/192 | 1.23 (0.37-4.10) | 0.52 |
| Smoker | 8/112 | 25/107 | 3.30 (1.42-7.66) |  |  | 20/233 | 34/221 | 1.91 (1.06-3.44) |  |
| Smoking level, pack-years | |  |  |  |  |  |  |  |  |
| ≥ 24 | 1/45 | 14/44 | 16.90 (2.10-136.25) | 0.022 |  | 12/115 | 18/109 | 1.72 (0.78-3.81) | 0.53 |
| < 24 | 7/67 | 11/63 | 1.62 (0.58-4.57) |  |  | 8/118 | 16/112 | 2.54 (1.02-6.34) |  |
| Drinking status |  |  |  |  |  |  |  |  |  |
| Nondrinker | 16/263 | 33/277 | 1.96 (1.05-3.66) | 0.23 |  | 21/357 | 26/339 | 1.31 (0.72-2.40) | 0.059 |
| Drinker | 6/110 | 23/113 | 3.89 (1.52-9.97) |  |  | 4/77 | 14/74 | 4.37 (1.36-14.04) |  |
| Family history |  |  |  |  |  |  |  |  |  |
| Negative | 16/280 | 44/278 | 2.73 (1.50-4.97) | 0.49 |  |  |  |  |  |
| Positive | 6/93 | 12/112 | 1.79 (0.64-4.99) |  |  |  |  |  |  |
| Nationality |  |  |  |  |  |  |  |  |  |
| Han | 20/349 | 53/362 | 2.61 (1.53-4.48) | 0.41 |  |  |  |  |  |
| Non-Han | 2/24 | 3/28 | 1.12 (0.17-7.45) |  |  |  |  |  |  |

Abbreviation: OR, odds ratio; CI, confidence interval.

^a^Number of genotype in N3/number of genotype in N0 + N1 + N2.

^b^In the Guangxi population, the ORs, 95% CIs and *P* values were calculated for N3 vs. N0 + N1 + N2 and adjusted for sex, age, status of smoking and drinking, smoking level, family history and nationality. In the Guangdong population, the ORs, 95% CIs and *P* values were calculated for N3 vs. N0 + N1 + N2 and adjusted for sex, age, status of smoking and drinking, and smoking level.

^c^For difference of ORs within each stratum.

**Supplementary Table 8**. Correlation between protein expression levels of AGO2 and rs3928672 genotypes in NPC tissues and non-cancerous nasopharyngeal tissues by immunohistochemistry

| **Tissues** | **Genotypes** | **Expression levels^a^, n** | | |  | ***P* value^b^** | |
| --- | --- | --- | --- | --- | --- | --- | --- |
|  |  | **Negative** | **Low** | **High** |  | **NPC vs. non-NPC** | **GA + AA vs. GG** |
| NPC tissues^c^ |  | 0 | 5 | 32 |  | 0.011 | 0.041 |
|  | GG | 0 | 3 | 10 |  |  |  |
|  | GA | 0 | 2 | 19 |  |  |  |
|  | AA | 0 | 0 | 3 |  |  |  |
| Non-cancerous^d^ |  | 0 | 8 | 10 |  |  | 0.34 |
|  | GG | 0 | 3 | 3 |  |  |  |
|  | GA | 0 | 2 | 2 |  |  |  |
|  | AA | 0 | 0 | 1 |  |  |  |
|  | NA^e^ | 0 | 3 | 4 |  |  |  |

^a^Expression levels were classified into three groups (negative, 0; low expression, score 2-4; high expression, score 5-8) with scores of the immunohistochemistry signals.

^b^The difference of the AGO2 protein level between the NPC tissues and non-cancerous nasopharyngeal tissues was assessed by a Wilcoxon signed-ranks test. The differences of the AGO2 protein level between the genotypes was assessed by logistic regression analysis.

^c^Nasopharyngeal carcinoma.

^d^Non-cancerous nasopharyngeal tissues.

^e^Not available duo to lack of blood DNA.

**Supplementary Table 11. Summary of previous studies on the genetic associations between *AGO2* and cancers**

| **Author (publication year)** | **Cancer type** | **Population** | **Cases/controls** | **SNP** | **Region** | **Allele^a^** | **Phenotype** | **Genetic model** | **OR / *P* value** |
| --- | --- | --- | --- | --- | --- | --- | --- | --- | --- |
| **Horikawa, Y., et al. (2008)** | Renal cell carcinoma | Caucasian | 279/278 | rs4961280 | Promoter | C/A | Occurrence | Dominant | 0.89 / >0.05 |
| **Yang, H., et al. (2008)** | Bladder cancer | Caucasian | 746/746 | rs4961280 | Promoter | C/A | Occurrence | Dominant | 1.00 / 0.98 |
| **Ye, Y., et al. (2008)** | Esophageal cancer | Caucasian | 346/346 | rs4961280 | Promoter | C/A | Occurrence | Dominant | 1.30 / 0.19 |
|  | Esophageal adenocarcinoma | Caucasian | 296/346 | rs4961280 | Promoter | C/A | Occurrence | Dominant | 1.18 / 0.343 |
| **Lin, J., et al. (2010)** | Renal cell carcinoma | Caucasian | 316 | rs4961280 | Promoter | C/A | Overall survival | Dominant | 1.15 / 0.644 |
|  |  |  |  | rs4961280 | Promoter | C/A | Recurrence | Recessive | 2.74 / 0.369 |
| **Kim, J. S., et al. (2010)** | Lung cancer | Korea | 552/552 | rss4965280 | Promoter | C/A | Occurrence | Additive | NA / 0.32 |
| **Sung, H., et al. (2011)** | Breast cancer | Korea | 559/567 | rs2292779 | Intron | G/C | Occurrence | Recessive | 1.24 / 0.22 |
|  |  |  |  | rs3864659 | Intron | A/C | Occurrence | **Dominant** | **0.67 / 0.03** |
|  |  |  |  | rs7016981 | Intron | T/C | Occurrence | Dominant | 1.22 / 0.22 |
|  |  |  |  | rs7824304 | Intron | C/T | Occurrence | Dominant | 0.68 / 0.06 |
|  |  |  |  | rs11786030 | 3’UTR | A/G | Occurrence | Recessive | 1.11 / 0.44 |
| **Permuth-Wey, J., et al. (2011)** | Epithelial ovarian cancer | Caucasian | 1815/1900 (Stage 1) | rs10088596 | intron | G/A | Occurrence | Dominant | 1.02 / 0.74 |
|  |  |  |  | rs10096598 | intron | A/G | Occurrence | Dominant | 0.8 / 0.114 |
|  |  |  |  | rs11786030 | intron | A/G | Occurrence | Dominant | 1.04 / 0.621 |
|  |  |  |  | rs11996715 | 5’UTR | A/C | Occurrence | **Dominant** | **0.85 / 0.043** |
|  |  |  |  | rs13276958 | intron | A/G | Occurrence | Dominant | 0.95 / 0.535 |
|  |  |  |  | rs1878478 | intron | A/G | Occurrence | Dominant | 0.86 / 0.403 |
|  |  |  |  | rs2176397 | intron | G/A | Occurrence | **Dominant** | **0.72 / 0.008** |
|  |  |  |  | rs2271738 | coding | G/A | Occurrence | Dominant | 0.95 / 0.493 |
|  |  |  |  | rs2280833 | intron | A/G | Occurrence | Dominant | 0.97 / 0.594 |
|  |  |  |  | rs2292775 | intron | G/A | Occurrence | Dominant | 2.49 / 0.206 |
|  |  |  |  | rs2292780 | intron | G/A | Occurrence | Dominant | 3.82 / 0.103 |
|  |  |  |  | rs2292781 | coding | G/A | Occurrence | Dominant | 0.96 / 0.563 |
|  |  |  |  | rs2944755 | intron | G/A | Occurrence | **Dominant** | **0.77 / 0.031** |
|  |  |  |  | rs2944765 | intron | A/C | Occurrence | Dominant | 0.94 / 0.414 |
|  |  |  |  | rs2977469 | intron | A/G | Occurrence | Dominant | 1.1 / 0.199 |
|  |  |  |  | rs2977477 | intron | G/A | Occurrence | Dominant | 1.01 / 0.91 |
|  |  |  |  | rs2977490 | intron | G/A | Occurrence | Dominant | 1.14 / 0.105 |
|  |  |  |  | rs3802214 | intron | G/A | Occurrence | Dominant | 1.07 / 0.363 |
|  |  |  |  | rs3864659 | intron | A/C | Occurrence | **Dominant** | **2.53 / 0.003** |
|  |  |  |  | rs3889488 | intron | A/G | Occurrence | Dominant | 0.8 / 0.102 |
|  |  |  |  | rs3928672 | intron | G/A | Occurrence | Dominant | 0.98 / 0.901 |
|  |  |  |  | rs6983924 | intron | A/G | Occurrence | Dominant | 1.06 / 0.39 |
|  |  |  |  | rs7001653 | intron | G/A | Occurrence | Dominant | 0.89 / 0.16 |
|  |  |  |  | rs7009635 | intron | G/A | Occurrence | Dominant | 0.94 / 0.468 |
|  |  |  |  | rs7824304 | intron | G/A | Occurrence | Dominant | 0.9 / 0.307 |
|  |  |  |  | rs7825416 | intron | G/A | Occurrence | Dominant | 0.74 / 0.158 |
|  |  |  |  | rs7832054 | intron | C/A | Occurrence | Dominant | 0.78 / 0.477 |
|  |  |  |  | rs7843258 | intron | G/A | Occurrence | Dominant | 1.07 / 0.365 |
|  |  |  |  | rs9694342 | intron | G/A | Occurrence | Recessive | 0.92 / 0.412 |
|  |  |  | 3987/4952 (Stage 2) | rs11996715 | 5’UTR | A/C | Occurrence | Dominant | NS^b^ |
|  |  |  |  | rs2176397 | intron | G/A | Occurrence | Dominant | NS^b^ |
|  |  |  |  | rs2944755 | intron | G/A | Occurrence | Dominant | NS^b^ |
|  |  |  |  | rs3864659 | intron | A/C | Occurrence | Dominant | NS^b^ |
| **Sung, H., et al. (2012)** | Breast cancer | Korea | 488 | rs2292779 | intron | C/G | Disease-free survival | **Additive** | **1.42 / 0.021** |
|  |  |  |  | rs3864659 | intron | A/C | Disease-free survival | Additive | 0.85 / 0.619 |
|  |  |  |  | rs7016981 | intron | T/C | Disease-free survival | Additive | 0.77 / 0.326 |
|  |  |  |  | rs7824304 | intron | C/T | Disease-free survival | Additive | 0.53 / 0.12 |
|  |  |  |  | rs11786030 | 3’UTR | A/G | Disease-free survival | **Dominant** | **2.62 / 0.002** |
|  |  |  |  | rs2292779 | intron | C/G | Overall survival | **Additive** | **2.94 / 0.001** |
|  |  |  |  | rs3864659 | intron | A/C | Overall survival | Additive | 1.11 / 0.825 |
|  |  |  |  | rs7016981 | intron | T/C | Overall survival | Additive | 1.48 / 0.254 |
|  |  |  |  | rs7824304 | intron | C/T | Overall survival | Additive | 0.59 / 0.34 |
|  |  |  |  | rs11786030 | 3’UTR | A/G | Overall survival | **Dominant** | **2.41 / 0.037** |
| **Weng, Y., et al. (2013)** | Malignant peripheral nerve sheath tumor | Chinese | 156/200 | rs7005286 | intron | C/T | Occurrence | **Additive** | **0.48 / 3.46×10^-4^** |

Abbreviation: OR, odds ratio; NA, not available; NS, not significant.

^a^Major allele/minor allele in the control population.

^b^In stage 2 replication (3987 epithelial ovarian cancer cases and 4952 controls), these SNPs (rs11996715, rs2176397, rs2944755 and rs3864659) were not significantly associated with epithelial ovarian cancer.

**References**

1. Ma F, Zhang H, Zhai Y, Huang W, Zhao C, Ou S, Zhou H, Yuan W, Wang Z, Wang H *et al*: **Functional polymorphism -31C/G in the promoter of BIRC5 gene and risk of nasopharyngeal carcinoma among chinese**. *PLoS One* 2011, **6**(2):e16748.

2. Zhang Y, Zhang H, Zhai Y, Wang Z, Ma F, Wang H, Li P, Yu L, Cui Y, He F *et al*: **A functional tandem-repeats polymorphism in the downstream of TERT is associated with the risk of nasopharyngeal carcinoma in Chinese population**. *BMC Med* 2011, **9**:106.

3. Horikawa Y, Wood CG, Yang H, Zhao H, Ye Y, Gu J, Lin J, Habuchi T, Wu X: **Single nucleotide polymorphisms of microRNA machinery genes modify the risk of renal cell carcinoma**. *Clin Cancer Res* 2008, **14**(23):7956-7962.

4. Yang H, Dinney CP, Ye Y, Zhu Y, Grossman HB, Wu X: **Evaluation of genetic variants in microRNA-related genes and risk of bladder cancer**. *Cancer Res* 2008, **68**(7):2530-2537.

5. Ye Y, Wang KK, Gu J, Yang H, Lin J, Ajani JA, Wu X: **Genetic variations in microRNA-related genes are novel susceptibility loci for esophageal cancer risk**. *Cancer Prev Res (Phila)* 2008, **1**(6):460-469.

6. Lin J, Horikawa Y, Tamboli P, Clague J, Wood CG, Wu X: **Genetic variations in microRNA-related genes are associated with survival and recurrence in patients with renal cell carcinoma**. *Carcinogenesis* 2010, **31**(10):1805-1812.

7. Kim JS, Choi YY, Jin G, Kang HG, Choi JE, Jeon HS, Lee WK, Kim DS, Kim CH, Kim YJ *et al*: **Association of a common AGO1 variant with lung cancer risk: a two-stage case-control study**. *Mol Carcinog* 2010, **49**(10):913-921.

8. Sung H, Lee KM, Choi JY, Han S, Lee JY, Li L, Park SK, Yoo KY, Noh DY, Ahn SH *et al*: **Common genetic polymorphisms of microRNA biogenesis pathway genes and risk of breast cancer: a case-control study in Korea**. *Breast Cancer Res Treat* 2011, **130**(3):939-951.

9. Permuth-Wey J, Kim D, Tsai YY, Lin HY, Chen YA, Barnholtz-Sloan J, Birrer MJ, Bloom G, Chanock SJ, Chen Z *et al*: **LIN28B polymorphisms influence susceptibility to epithelial ovarian cancer**. *Cancer Res* 2011, **71**(11):3896-3903.

10. Sung H, Jeon S, Lee KM, Han S, Song M, Choi JY, Park SK, Yoo KY, Noh DY, Ahn SH *et al*: **Common genetic polymorphisms of microRNA biogenesis pathway genes and breast cancer survival**. *BMC Cancer* 2012, **12**:195.

11. Weng Y, Chen Y, Chen J, Liu Y, Bao T: **Common genetic variants in the microRNA biogenesis pathway are associated with malignant peripheral nerve sheath tumor risk in a Chinese population**. *Cancer Epidemiol* 2013, **37**(6):913-916.
